# AI driven analysis of MRI to measure health and disease progression in FSHD

**DOI:** 10.1038/s41598-024-65802-x

**Published:** 2024-07-05

**Authors:** Lara Riem, Olivia DuCharme, Matthew Cousins, Xue Feng, Allison Kenney, Jacob Morris, Stephen J. Tapscott, Rabi Tawil, Jeff Statland, Dennis Shaw, Leo Wang, Michaela Walker, Leann Lewis, Michael A. Jacobs, Doris G. Leung, Seth D. Friedman, Silvia S. Blemker

**Affiliations:** 1 Springbok Analytics, 110 Old Preston Ave., Charlottesville, VA 22902 USA; 2 Seattle Children’s Hospital, Seattle, WA USA; 3 Kennedy Krieger Institute, Baltimore, MD USA; 4 Fred Hutchinson Cancer Center, Seattle, WA USA; 5 University of Washington, Seattle, WA USA; 6 University of Rochester Medical Center, Rochester, NY USA; 7 University of Kansas Medical Center, Kansas City, KS USA; 8 University of Texas Health Science Center at Houston (UTHealth Houston), Houston, TX USA; 9 Johns Hopkins University School of Medicine, Baltimore, MD USA; 10 Rice University, Houston, TX USA; 11 University of Virginia, Charlottesville, VA USA

**Keywords:** MRI, FSHD, Fat fraction, Muscle volume, Progression, Atrophy, Volumetric analysis, Neuromuscular disease, Skeletal muscle, Biomedical engineering

## Abstract

Facioscapulohumeral muscular dystrophy (FSHD) affects roughly 1 in 7500 individuals. While at the population level there is a general pattern of affected muscles, there is substantial heterogeneity in muscle expression across- and within-patients. There can also be substantial variation in the pattern of fat and water signal intensity within a single muscle. While quantifying individual muscles across their full length using magnetic resonance imaging (MRI) represents the optimal approach to follow disease progression and evaluate therapeutic response, the ability to automate this process has been limited. The goal of this work was to develop and optimize an artificial intelligence-based image segmentation approach to comprehensively measure muscle volume, fat fraction, fat fraction distribution, and elevated short-tau inversion recovery signal in the musculature of patients with FSHD. Intra-rater, inter-rater, and scan-rescan analyses demonstrated that the developed methods are robust and precise. Representative cases and derived metrics of volume, cross-sectional area, and 3D pixel-maps demonstrate unique intramuscular patterns of disease. Future work focuses on leveraging these AI methods to include upper body output and aggregating individual muscle data across studies to determine best-fit models for characterizing progression and monitoring therapeutic modulation of MRI biomarkers.

## Introduction

Facioscapulohumeral muscular dystrophy (FSHD) is a slowly progressing muscle disease related to toxic expression of the protein DUX4 affecting ~ 1 in 7500 individuals^1^. DUX4 expression and histologic changes are primary measures of disease progression and have been employed in early treatment trials of FSHD; however, these measures require muscle biopsies, which are invasive and provide only limited samples^2–4^. We previously showed that, while needle biopsies provide important insight into DUX4 level and disease activity, they do not provide a complete picture of disease state in each muscle. For example, biopsies from central regions of the tibialis anterior muscle that appear normal on MRI (no evidence of fat or short tau inversion recovery (STIR) bright signal) often show elevated levels of DUX4 if there is evidence of disease (fat infiltration/inflammation) somewhere along the individual muscle extent^4^. This finding supports the need to develop an analytic method that can efficiently quantify tissue state (fat, normal intensity muscle, muscle with STIR hyperintensity) throughout the entirety of each individual muscle. Such a method would provide complete risk profiles for individual muscles that can be monitored over time.

Disease progression over time typically occurs over years in adult-onset FSHD^5–13^ and can affect skeletal muscles across the entire body. While specific muscles (e.g. scapula fixation muscles, hamstrings, tibialis anterior, and gastrocnemius) are routinely affected when summarizing FSHD cohorts, there is significant patient-to-patient heterogeneity in disease expression and the involvement of other muscles. This variation includes side-to-side asymmetry, as well as unique patterns and potentially different progression rates within individual muscles, and the relationships between these features and other disease biomarkers (e.g. allele length, methylation status, DUX4 levels) likely have pathophysiologic significance and could provide further insights into the phenotype of this disease^5–8,10–13^. MRI has emerged as the gold standard method for visualizing patterns of muscle involvement in FSHD. Despite the relative ease in acquiring whole body imaging data, analytic methods that isolate individual muscles into accurate units remain limited, which has consequently hindered the use of MRI as an outcome measure in natural history studies and clinical trials. One of the major bottlenecks in developing MRI-based biomarkers has been the process of segmenting individual muscles. For studies surveying large regions, qualitative rating scales can be used to efficiently generate heat maps^12,14^. However, qualitative rating scales divide the spectrum of disease into a few large strata, which makes them less useful as short-term biomarkers that need to be sensitive to change. Quantification methods emphasizing full-capture muscles have focused on composite muscle regions^15^ or analyzing central slices of focused anatomies (e.g. thigh/calf) to shorten the process of manually segmenting muscles and to simplify computational demands^13,16,17^. While some quantitative methods have been developed to efficiently parcel individual muscles within central regions from leg anatomy^18,19^, the development of automated tools to measure individual muscles across their full anatomical extent is more challenging and has not been demonstrated in FSHD to date.

In parallel to these efforts, our group has pioneered the development of AI-based muscle segmentation in a range of pathologies, muscle morphology, and imaging sequences^20–23^. The result is a highly efficient AI segmentation process that can produce consistent full-coverage output. By using these methods to analyze scans of FSHD patients, we aimed to develop a suite of targeted fat and inflammatory measures that could be widely applied to existing data and future therapeutic trials.

The overall goal of this work is to leverage our AI-based image segmentation to validate a detailed, muscle-level suite of metrics for the fast and accurate assessment of fat (using T1 Dixon) and free water content (using STIR) in lower limb muscles in patients with FSHD. These metrics include individual muscle-level volume quantification, individual muscle-level fat fraction quantification, distribution of fat fraction throughout each muscle, and quantification of STIR hyperintensity within each muscle. Specifically, we (i) refined the segmentation process to be effectively applied to MR images collected in patients with FSHD, (ii) analyzed two retrospective cohorts to test the intra-observer, inter-observer, and scan-rescan variability in the measures, (iii) compared the muscle-derived metrics with clinical assessments, and (iv) present example applications of the novel metrics that will be examined in future studies. By performing this detailed characterization of FSHD MRI scans, we seek to identify the salient imaging features that can be used to detect disease progression and to optimize protocols for collecting imaging biomarkers so they can be incorporated into future clinical trials.

## Methods

### Dataset overview

We leveraged multiple retrospective datasets to develop and validate the AI-based muscle-level FSHD analyses. The datasets included lower extremity MRI scans collected from 58 patients with FSHD from four different sites as part of two studies (Wellstone cohort N = 34^4^, µ = 47 years, Male = 16/Female = 18, Clinical Severity Score (CSS) µ = 4—Seattle Children’s, University of Kansas, University of Rochester; Kennedy Krieger Institute (KKI) cohort N = 30, µ = 50 years, Male = 12/Female = 18, CSS µ = 6—KKI/Johns Hopkins University). All experimental protocols used to collect the data were in accordance with relevant guidelines/regulations and were approved by local or central IRBs at Seattle Children’s Hospital, University of Kansas, University of Rochester, and KKI. Subjects provided informed consent for data-collection and aggregation, consistent with the local or central IRBs at all the same institutions. The scans varied slightly in lower-body coverage depending on the acquisition method: Wellstone scans covered muscles of the thigh and calf (n = 34), while the KKI scans covered muscles of the hip and thigh with no coverage of the calf (n = 30). In 26 of the 30 patients from KKI, we analyzed follow-up scans that were collected three months after the initial scans; these analyses served as a scan-rescan test for muscle analysis metrics. In 18 patients from the Wellstone cohort and 3 patients from KKI, we analyzed follow up scans that were collected 12 months from the initial scans; these analyses served to generate example cases for comparing volume to CSA to pixel-analyses after one year of disease progression.

### Imaging protocols

For the Wellstone subjects, two-point Dixon (TE = 1.35/2.58 ms; TR = 4.12 ms, 3 mm slices) and STIR (TE = 38 ms, TR = 5000 ms, 5 mm slices) scans were collected on Siemens PRISMA scanners with coverage from the distal end of the pelvis through the patient’s ankles. At KKI, two-point Dixon (TE = 3.69/4.92 ms; TR = 150 ms, 5 mm slices) and STIR (TE = 91 ms, TR = 1800, 5 mm slices) images were collected on a Siemens PRISMA scanner with coverage from just above the clavicle to just below the knee.

### AI-based muscle segmentation

We utilized an AI-based approach similar to our previously published algorithm^22^ to segment the boundaries of up to 36 muscles (depending on coverage) of the lower extremity from the Dixon water images. As a brief description, the AI model utilized a modified 3D U-Net structure. Specifically, every level in the encoder contains layers of two blocks of a 3 × 3 × 3 convolution layer, a batch normalization (BN) layer, and a rectified linear unit (ReLU) activation layer, followed by a 2 × 2 × 2 maxpooling, excluding the bottom-most level. In the decoder, each level consists of layers with a 2 × 2 × 2 deconvolution layer, followed by two blocks of a 3 × 3 × 3 convolution, a BN, and a ReLU layer. In addition, feature maps from the encoder were concatenated to those of the same resolution in the decoder as the skip connection. The final block of the network contains a 1 × 1 × 1 convolution layer to reduce the dimension of the features to match the number of label maps, followed by a pixelwise softmax classifier. The algorithms were implemented based on the framework and training of TensorFlow. During training, weights were initialized randomly from Gaussian distribution and updated, with an initial learning rate of 0.01 and the pixelwise dice loss + cross-entropy as the loss function, using the adaptive moment estimation (Adam) optimizer for gradient descent. The initial learning rate was 0.01 and the loss function was pixelwise dice loss + cross entropy + volume error. For a detailed description of our AI model, please see our previous publication^22^.

The AI model used in this study was trained on 809 scans. These scans included lower extremity images collected on individuals from a range of demographics and scans acquired using a range of settings. Individuals included athletes of varying sports and performance levels, healthy adults, and patients with FSHD (99 specifically). The scan protocols included images acquired using two-point Dixon, T1-weighted, and proton density images on multiple scanners. To further increase the effectiveness of AI training, extensive data augmentation, including shearing and rotation, was applied in the training process. The diversity in the training data set resulted in an AI algorithm that could be generalized to the four-site dataset used for this study. The AI algorithm was validated by comparison of AI-based segmentation with vetted segmentations on several scans of varying fat fraction levels that were not included in the training set.

**Figure 1:**
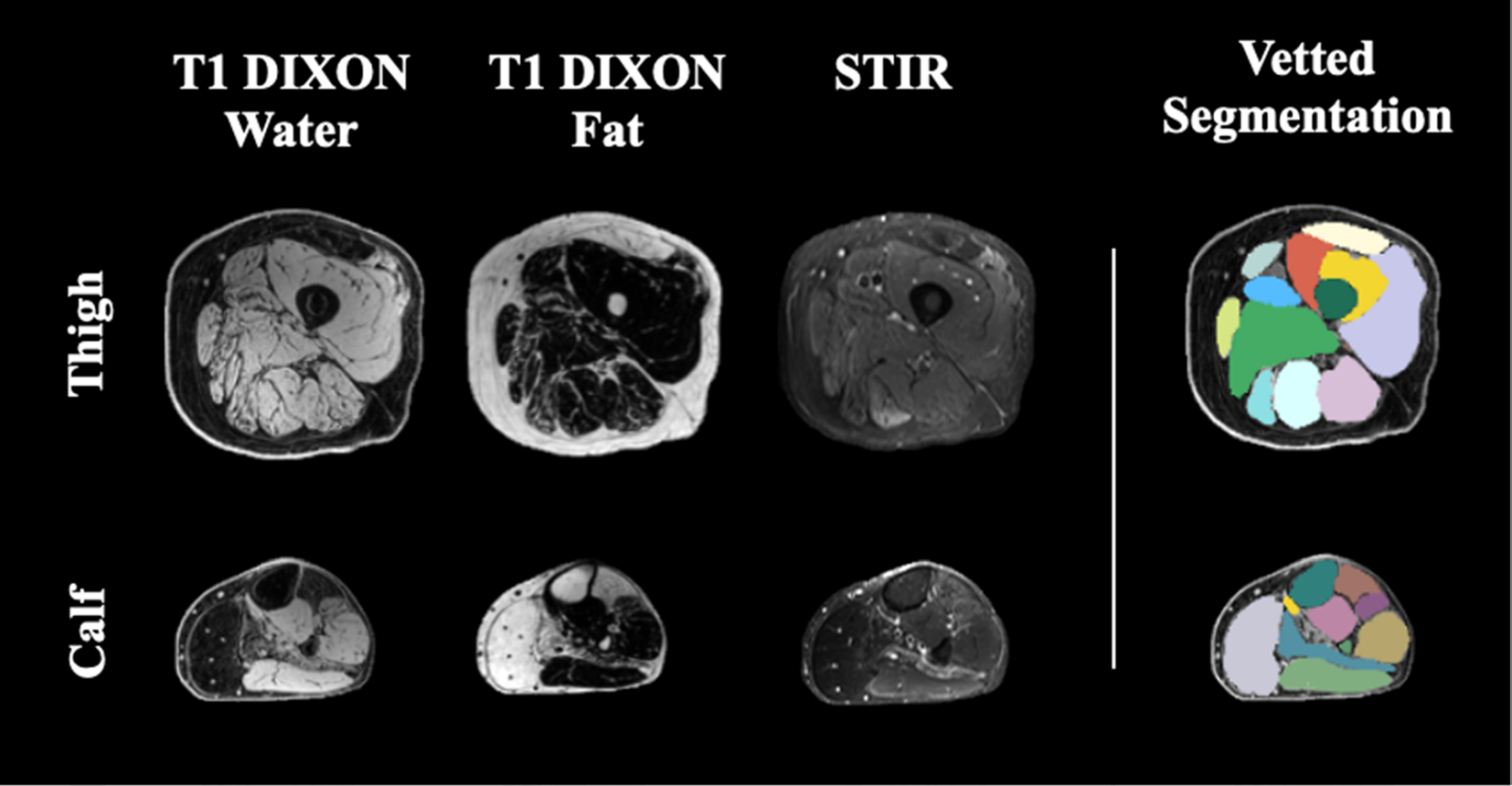
Example of images and segmentations as used in this study. All patients had Dixon T1 water and fat phase images, while Wellstone patients had STIR images as well. Final vetted musculature labels are also displayed for a few example slices.

MRI scans were preprocessed (registration, inhomogeneity correction, signal-normalization) as per our published methods^22^ to produce continuous, axial 3D T1 Dixon water and fat images for all 64 patients and their respective follow up scans. For the T1 Dixon water component images, the boundaries of up to thirty-six muscles in both lower extremity limbs were segmented via the 3D AI algorithm. At the end of processing, 3D images with matching dimensions/coverage were generated for each subject: (1) T1 Dixon water component image, (2) T1 Dixon fat component image, and (3) final muscle label map (Fig. 1).

For each AI output, a trained segmentation engineer manually reviewed and edited for accuracy (process we call “vetting”) utilizing the paint tool in 3D Slicer (v4.11). This stage required between one and three hours, depending on the level of fatty replacement (with higher fatty replacement muscles requiring more user interaction time for verifying contours). The data set used to test the AI output, a single researcher evaluated and vetted/refined all segmentations.

Due to the varying scan coverage between sites, the muscles that were fully captured varied across sites (A: Wellstone, B: KKI): quadratus lumborum (B), psoas major (B), iliacus (B), gluteus medius (B), gluteus maximus (B), gluteus minimus (B), piriformis (B), gemelli (B), quadratus femoris (B), obturator internus (B), obturator externus (B), pectineus (B), tensor fasciae latae (B), rectus femoris (A,B), vastus lateralis (A,B), vastus intermedius (A,B), vastus medialis (A,B), sartorius (A,B), adductor brevis (A,B), adductor magnus (A,B), adductor longus (A,B), gracilis (A,B), semitendinosus (A,B), semimembranosus (A,B), biceps femoris: long head (A,B), biceps femoris: short head (A,B), popliteus (A), gastrocnemius: medial head (A), gastrocnemius: lateral head) (A), soleus (A), tibialis anterior (A), phalangeal extensors (A), fibularis muscles (including the fibularis longus, fibularis brevis, and fibularis tertius) (A), tibialis posterior (A), flexor digitorum longus (A), flexor hallucis longus (A). These differences in the muscles analyzed was due to the differing anatomical coverage between the two cohorts. The integration of these Dixon datasets was used for reliability measures. For test–retest of derived values, KKI 3-month data was processed similarly. For longer-term follow-up, the scans from Wellstone and KKI subjects followed at 12 months were analyzed to generate volume, surface area, and pixel data to demonstrate longitudinal evaluation of muscle and fat metrics using this algorithm.

### General approach for examining consistency and precision

The consistency and precision of the muscle-by-muscle 3D segmentations was examined in four ways. First, the short-term stability of the measurements was tested by analyzing scans collected within three months timeframe (KKI, n = 26 FSHD scans). Three to four months has been previously considered a short enough timeframe that disease progression is expected to be unlikely in most FSHD patients^7,11,24^. Second, we selected three scans to perform both inter-observer (two different engineers processed the same scans) and (third) intra-observer (the same engineer processed the scans twice) analyses. To evaluate the method across a representative range of possible datasets, the three selected scans included: one scan showing high disease progression (elevated fat infiltration throughout the lower limb musculature), one scan showing low disease progression (minimal fat infiltration throughout the lower limb musculature), and one with a medium level of disease progression. Dice similarity coefficients (Eq. 4) were calculated for each of these analyses, comparing each muscle and each set of segmentations^25^. Fourth, to validate the AI segmentation performance, the raw AI labeled output was compared to the vetted output (from the engineer who only vetted once). The same scans as utilized above were selected for this analysis, as (1) the AI was not trained on these scans previously, and (2) they represented scans of varying difficulty for the AI to segment.

1 $${Dice}_{RoI}=\frac{{Label\,Map\, 1}_{RoI} \cap {Label\, Map\, 2}_{RoI} }{{Label \,Map \,1}_{RoI} \cup {Label \,Map \,2}_{RoI}},$$

### Quantification of muscle volume and fat fraction

For each muscle, the boundary volume was calculated by summing the total number of pixels labeled for that segmented muscle and multiplied by the pixel’s voxel volume. The fat fraction (FF) (%) for each muscle was determined using the equation below:

2 $${Fat Fraction}_{muscle}={\sum }_{p=1}^{TP}\frac{{FSI}_{p}}{{FSI}_{p}+{WSI}_{p} }*\frac{1}{TP}*100,$$

where TP represents the total number of pixels within the muscle, p is an individual pixel, FSI is the pixel’s fat signal intensity and WSI is the pixel’s water signal intensity. Fat volume and lean muscle volumes were then calculated according to the following equations:

3 $${Fat\, Volume}_{muscle}={ Boundary \,Volume}_{muscle}*\frac{{Fat \,Fraction}_{muscle}}{100},$$

4 $${Lean\, Volume}_{muscle}={ Boundary\, Volume}_{muscle}*(1- \frac{{Fat \,Fraction}_{muscle}}{100}),$$

The precision of these calculations was assessed by extracting fat fraction and boundary volume from the three-month repeat, intra-observer, and inter-observer segmentations. The absolute volume error was found by calculating the absolute difference in volume for each repeat observation and dividing it by the average of the two observations. The absolute difference in FF was found by taking the absolute difference in FF between the two observations.

Given the slight chemical shift that occurs in the fat images acquired using Dixon MRI techniques, we examined the impact of eroding the segmentation to remove pixels on the border of the muscle boundary. Each muscle label was eroded on a slice-by-slice basis by a radial footprint of approximately 4 mm on both the baseline and 3-month scan. This approach led to erosion of each label by approximately one pixel. Then, the difference in fat fraction from baseline to 3-months, calculated from both original and eroded labels, was compared.

To assess how fat fractions measured by MRI relate to qualitative clinical measures, we compared the qualitative ratings of the tibialis anterior muscles of 34 FSHD patients with fat fractions calculated from the tibialis anterior muscles in the same subjects. Ratings were based on the published scales^26,27^, 1: normal appearance, 2: scattered small areas of abnormality, 3: numerous discrete areas of increased signal intensity, less than 30% of volume, 4: numerous discrete areas with early confluence, 30–60% of muscle volume, 5: > 60% replaced, patchy with loss of fascial structure; 6: complete fascial structure loss. Volumetric fat fractions calculated from the MRI were correlated (Spearman’s) to the clinical fat rating on all patients, including measurements for both left and right sides.

### Quantification of fat distribution within each muscle

**Figure 2:**
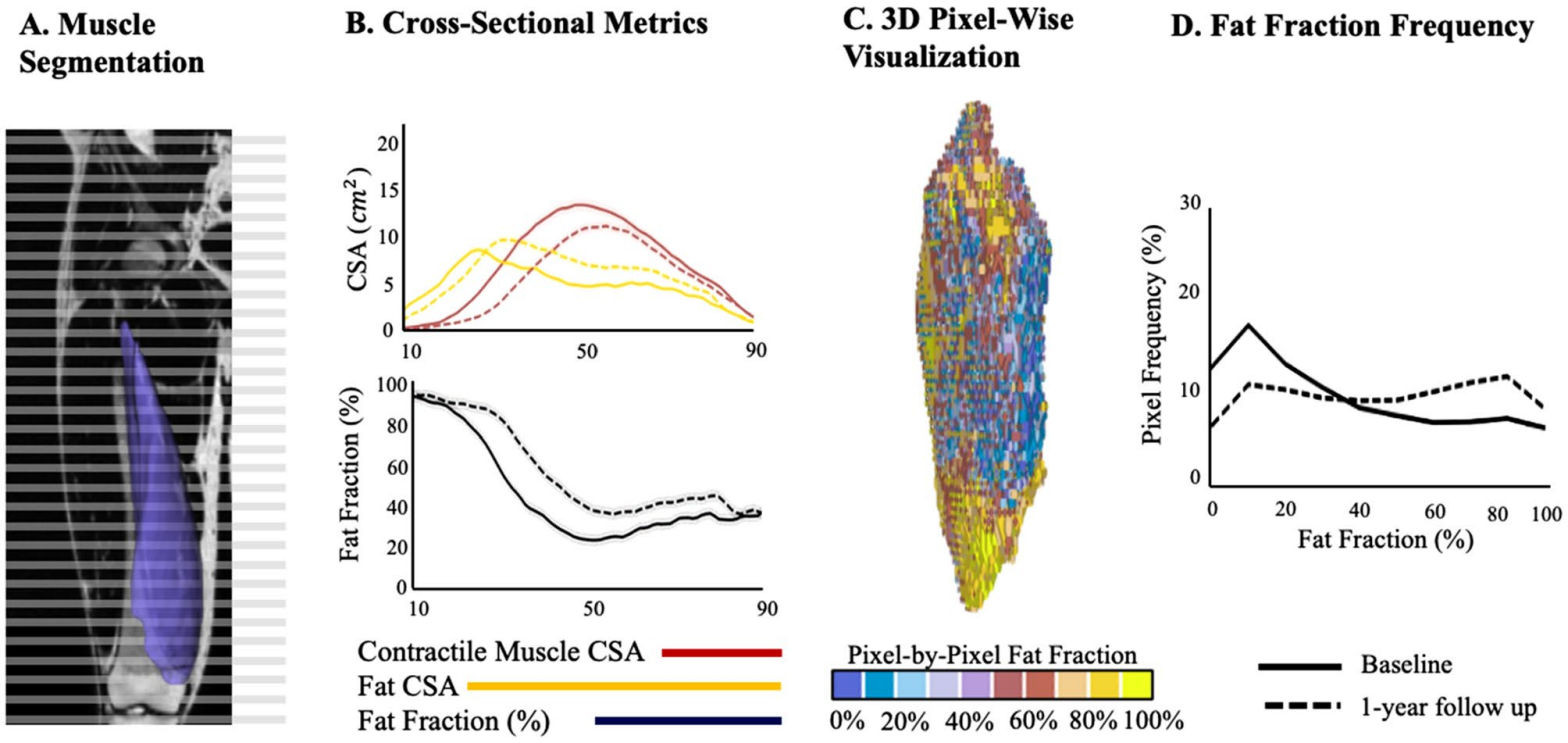
(**A**) Example of the process to analyze a muscle’s (vastus medialis) cross-sectional area (CSA) muscle composition from a 3D segmentation. (**B**) The muscle boundary CSA, contractile muscle CSA, fat CSA, and fat fraction (%) were found at each axial slice as a function of axial distance slice-by-slice moving inferior (distal) to superior (proximal). As the muscle characteristics were expressed as a function of axial slice location, the first and last slice’s axial location in which the muscle was present were recorded. Muscle characteristics were then expressed as a function of percent along muscle from 0% (first, inferior location muscle is present) to 100% (last, superior location muscle is present) and were interpolated between points by 1% increments. Shown is the final CSA from 10 to 90%, the last 10% ends were excluded from visualization as the CSA is so small on the ends it increases errors. (**C**) The 3D model demonstrated again, this time with pixel-by-pixel fat fraction, in which each pixel is color coded based on fat fraction (%) from 0% (blue) to 100% (yellow). (**D**) Lastly, the frequency in which each pixel’s fat fraction is found is displayed as a histogram from 0% fat infiltration to 100% fat infiltration at baseline (solid line) and 1 year later (dotted line).

Since disease progression is often heterogeneous within each muscle in FSHD, we incorporated methods for visualizing and quantifying the heterogeneity of fat fraction throughout each muscle (Fig. 2). First, we computed muscle and fat quantities within each axial slice of each muscle and displayed those metrics as a function of length along the muscle. The muscle boundary cross-sectional area (CSA), lean muscle CSA, fat CSA, and area fat fraction (%) were calculated at each axial slice. The measures were then plotted as a function of longitudinal distance slice-by-slice moving inferior (distal) to superior (proximal). Muscle boundary CSA was calculated by summing the total number of pixels labeled for that segmented muscle and multiplied by the pixel area. Area fat fraction was calculated using Eq. (2), with the modification that TP was restrained to the pixels in the slice of interest. Fat and lean muscle CSA were calculated using Eqs. (3 and 4), respectively, with the modification that CSA was used in place of volume. Muscle characteristics were then expressed as a function of the percentage of the muscle length from 0% (inferior end of the muscle) to 100% (superior end of the muscle) and were interpolated between slices by 1% increments. We also generated histograms of fat fraction to assess the overall composition of the muscle and 3D visualizations of fat fraction distribution within muscles. In the figures, each pixel is assigned a color based on its fat fraction, using a discrete colormap where 0% fat is blue, and 100% fat is yellow (Fig. 2C).

### Quantification of STIR brightness

**Figure 3:**
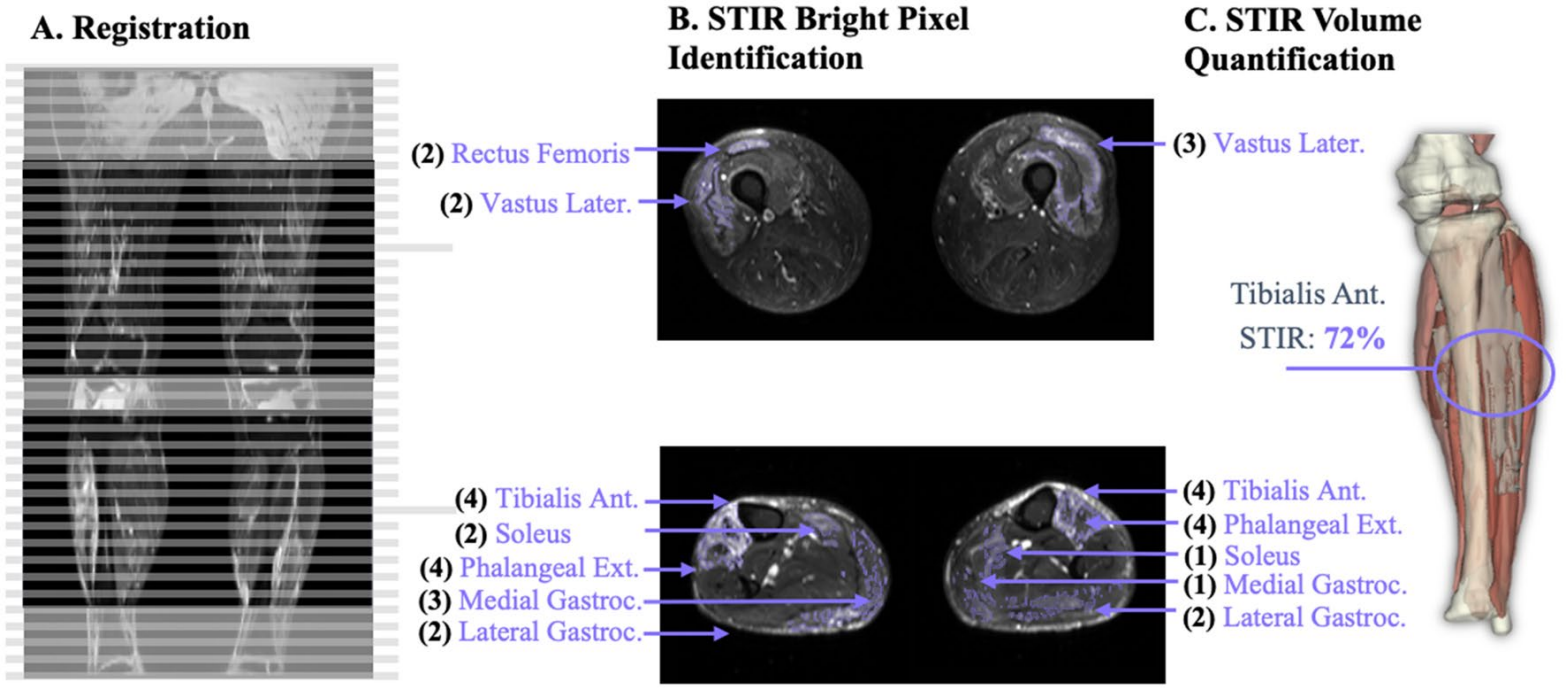
Overview of process to objectively quantify STIR content in individual muscles as shown through an example patient. (**A**) The DIXON images are registered to the STIR images (displayed in pane) to ensure the same coverage is used. (**B**) The STIR bright regions are segmented for every slice across all muscles from the STIR scans. The clinical STIR grading (number in black) and manually segmented STIR portion (in purple) for each muscle from the STIR image is demonstrated. (**C**) Final STIR percent is found by finding the content of labeled STIR present in the labeled muscle’s region. A 3D rendering of the overlaid segmented STIR (bright blue) and muscle (pinkish/red) is illustrated, in which a final STIR content of 72% was found for the tibialis anterior.

To develop and validate a method for automated quantification of STIR brightness (Fig. 3), we used datasets collected from 31 of the 34 Wellstone subjects at the baseline timepoint (3 patients did not have STIR images collected). These datasets included both T1 Dixon and STIR MRI scans. As described above, the T1 Dixon data included continuous axial images from the subject’s ankles to the distal edge of the pelvis. The STIR sequence was an axial scan split into two regions for the calf and thigh respectively, and only the middle portion of each anatomical region was acquired. One clinician (DS) graded the STIR presence in each individual muscle for each subject. For each muscle, STIR presence was graded on a scale from 0 to 4 as per our modified scale^28^ (0: normal appearance, 1: mild diffuse elevation, 2: moderate signal elevations, < 30% of the target volume, 3: moderate signal elevations, 30–60% of the target volume, 4: moderate signal elevations, > 60% of the target volume).

The STIR images were manually segmented by a trained segmentation engineer. The segmentation process included identifying if each pixel or region of pixels with relatively higher pixel intensity was considered “STIR positive” (STIR +). All other pixels were labeled as “STIR negative” (STIR-). The process for determining which pixels should be considered STIR positive was originally informed and checked by consultation with a trained radiologist. In the first step, engineers segmented brightly colored STIR + regions using the threshold image paint tool within slicer3D which operates^29^ on circular regions of up to 20% of the limb (to account for changes in image intensity throughout the image) slice by slice via an Otsu filter. If thresholded bright pixels were from a change in intensity not due to the change in pixel intensity seen between fat and contractile muscle tissue, it was segmented as STIR + (this was verified utilizing the DIXON water and fat images or with experience analyzing these images). Additionally, the shape of the thresholded pixels helped inform the segmentation engineers to common artifacts, in which very smooth regions often found at the edges of the limb due to image biasing were ignored. As result of this process, each STIR dataset included labels of STIR positive pixels. Once the manual segmentation approach was validated, we trained a deep convolutional neural network, using a process previously applied to the analysis of clinical rotator cuff images^30^, to provide an initial segmentation that could then be vetted and corrected by a trained segmentation engineer. We found that the addition of an AI-derived initial segmentation sped up the vetting time by roughly 50%.

To precisely overlay STIR positive labels and the vetted muscle labels from the Dixon scans, we completed rigid 3D registration of the muscle labels and Dixon images to the STIR image using Advanced Normalization Tools (ANTs)^31^. This algorithm transforms the Dixon image and muscle label data via rigid body translation and rotation to minimize the difference between the two datasets. This process ensured that any small changes to subject position and/or STIR coverage were accounted for and the transformed Dixon-based muscle labels matched the STIR images. Once transformed, the muscle labels were eroded to remove the outermost layer of segmented pixels to account for possible errors associated with the registration process. For each muscle, the quantified STIR content (%) was determined as the number of STIR positive pixels within the muscle boundary expressed as a percentage of the total number of pixels in the muscle boundary.

To evaluate the STIR quantification method, we performed a Spearman correlation analysis between the quantified STIR content (%) and clinical STIR grading (0–4). The analysis included the 20 muscles (bilaterally) that were consistently captured in the STIR images (rectus femoris, vastus lateralis, vastus intermedius, vastus medialis, sartorius, adductor brevis, adductor magnus, adductor longus, gracilis, semitendinosus, semimembranosus, biceps femoris: long head, biceps femoris: short head, gastrocnemius: medial head, gastrocnemius: lateral head, soleus, tibialis anterior, phalangeal extensors, fibularis muscles and tibialis posterior). We also assessed the repeatability of the STIR quantification by performing inter-observer and intra-observer analyses on 10 randomly selected datasets. Repeatability was evaluated by calculating the absolute difference in STIR content between the two observations.

## Results

### Evaluation of muscle boundary segmentation

**Figure 4:**
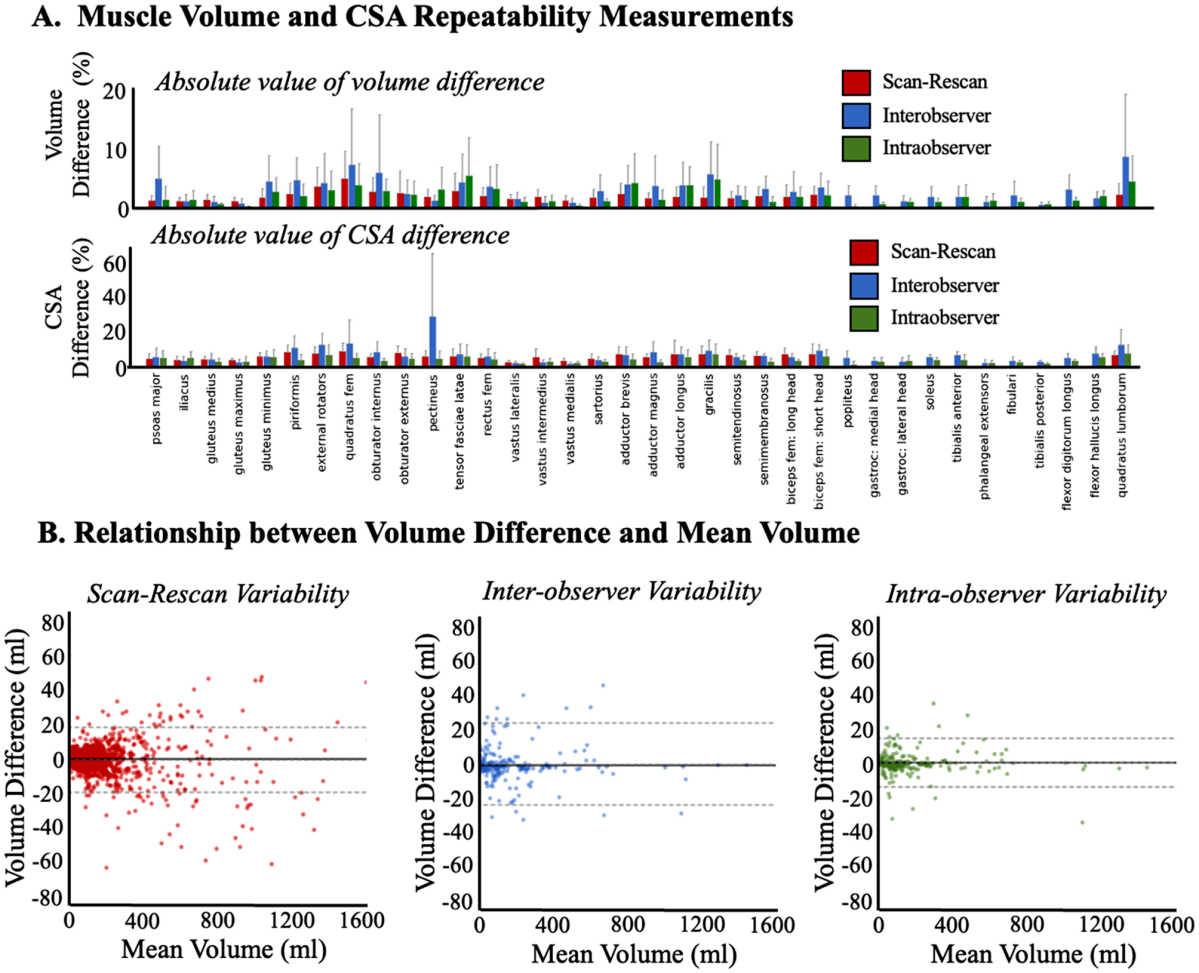
(**A**) Absolute volume error (top) and absolute cross-sectional area (CSA) error (bottom) for each individual muscle for the scan-rescan (red, n = 26), interobserver (blue, n = 3), and intraobserver (green, n = 3) volume variability. (**B**) Bland–Altman plots for scan-rescan (red, left), interobserver (blue, middle), and intraobserver (green, right) volume variability.

Overall, the AI-driven muscle boundary segmentation approach showed strong scan-rescan, inter-observer, intra-observer, and AI output-to-vetted reliability (Fig. 4; Supplemental Table 1, 2, and 3). The Dice similarity coefficient calculated from the intra- and inter-observer repeatability tests (Supplemental Table 2) ranged from 0.83 to 0.97. The Dice similarity coefficient calculated from comparing the raw AI labels to the vetted segmentations ranged from 0.65 ± 0.56 (pectineus) to 0.98 ± 0.02 (vastus medialis) and was most commonly above 0.90 (Supplemental Table 3). As expected, the Dice similarity coefficients were higher for the lower disease progression cases and lower for higher disease progression cases. Dice similarity coefficients between segmentations within the same observer were higher than between observers. These trends were also observed in the absolute volume error calculations (Fig. 4A, Supplemental Table 1). Average absolute volume error calculated from intra- and inter-observer tests varied from 0.16 ± 0.27% (intra-observer, popliteus) to 9.03 ± 13.82% (inter-observer; quadratus lumborum).The absolute volume errors when comparing the raw AI label to the vetted segmentations ranged from 1.15 ± 1.6% (vastus medialis) to 20.69 ± 25.44% (gracilis) and was most commonly less than 3% (Supplemental Table 3). Absolute volume errors between the three-month repeat scans also demonstrated consistency: the average absolute volume error ranged from 1.03 ± 0.77% (gluteus maximus) to 5.08 ± 5.05% (quadratus femoris). Bland–Altman analyses (Fig. 4B) demonstrated proportional bias for scan-rescan tests towards lower volumes at 3 months compared to baseline (t = − 2.77, df = 1299, *p* = 0.003). However, there was no bias in the inter-observer (t = − 0.74, df = 215, *p* = 0.229) or intra-observer (t = 0.60, df = 215, *p* = 0.120) comparisons.

### Evaluation of fat fraction measurements

**Figure 5:**
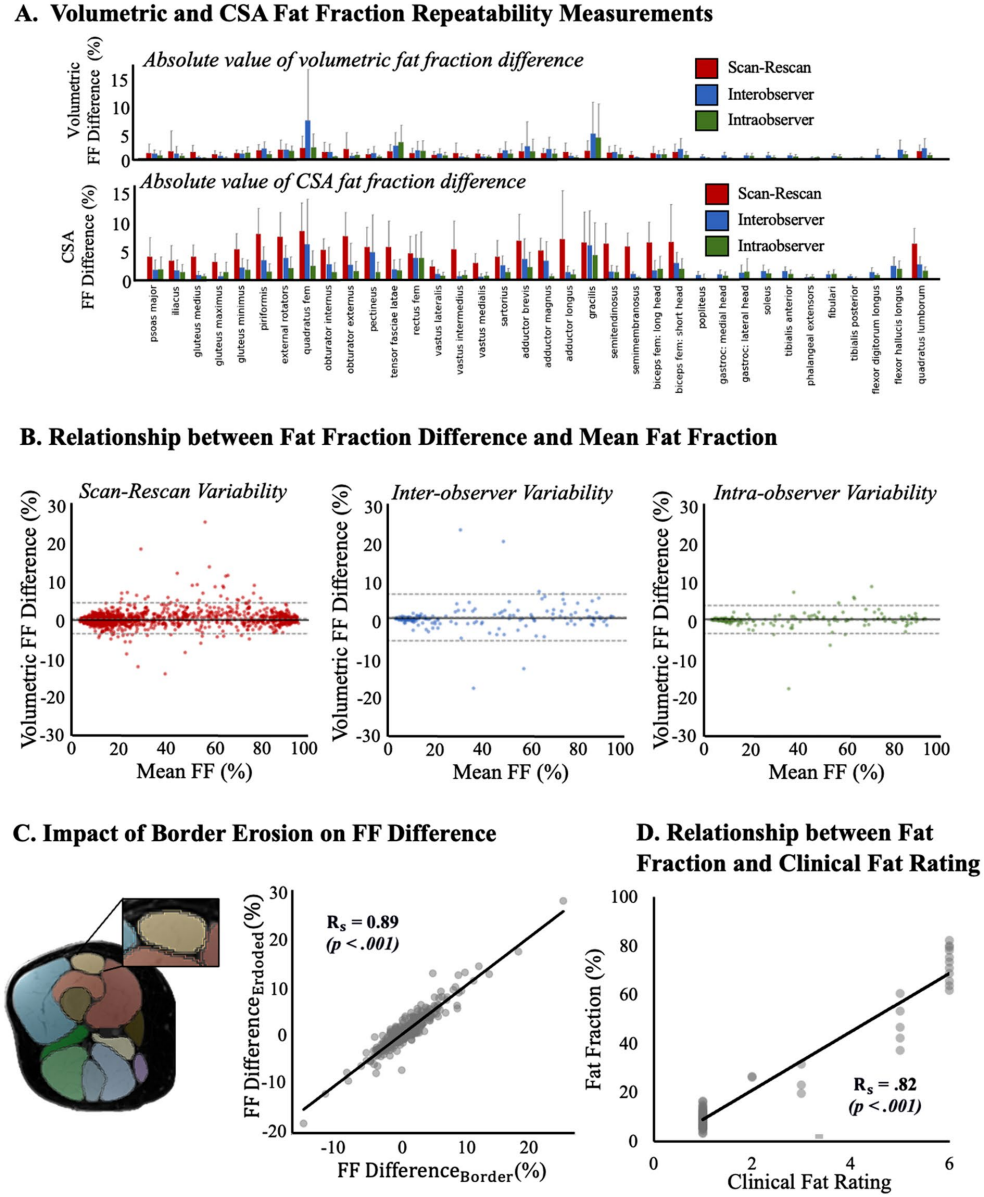
(**A**) Absolute fat fraction difference (top) and cross-sectional area (CSA) fat fraction difference (bottom) for each individual muscle for the scan-rescan (red, n = 26), interobserver (blue, n = 3), and intraobserver (green, n = 3) volume variability (**B**) Bland–Altman plots for scan-rescan (red, left), interobserver (blue, middle), and intraobserver (green, right) volumetric fat fraction difference. (**C**) Comparison of muscle labels from overall raw label (outline) and an eroded version of the label (filled) for the 26 KKI scan-rescan cases. Correlation of the difference in raw fat fraction difference (%) and eroded muscle fat fraction difference (%) for all complete coverage muscle. The linear regression line is shown in black (y = 1.03*x + 0.02). (**D**) Correlation between the clinical fat rating and fat fraction found for all Wellstone patients with complete coverage muscles.

Fat fractions calculated based on the muscle boundary segmentations also demonstrated strong consistency (Fig. 5A). The absolute fat fraction difference between the intra- and inter-observer analyses ranged from 0.06 ± 0.04% (intra-observer; tibialis posterior) to 7.19 ± 11.90% (intra-observer, quadratus femoris). The absolute fat fraction difference between the AI label and vetted segmentations ranged from 0.22 ± 0.22% (vastus medialis) to 20.62 ± 20.39% (gracilis). The fat fraction differences between the baseline and 3-month repeat scans ranged from 0.63 ± 0.66% (semimembranosus) to 1.90 ± 2.37% (quadratus femoris). Bland–Altman analyses (Fig. 5B) demonstrated proportional bias for scan-rescan tests towards higher fat fraction at 3 months compared to baseline (t = 6.124, df = 1299, *p* < 0.001) comparisons. However, there was no bias in the inter-observer (t = − 0.51, df = 215, *p* = 0.305) or intra-observer (t = 1.18, df = 215, *p* = 0.120). There was a high correlation between change in fat infiltration using the border (raw) labels and using the eroded labels (Fig. 5C, Rs = 0.90, *p* < 0.001). The average difference in fat infiltration between the two methods was 0.44% with a standard deviation of 0.63%. Lastly, clinical fat ratings strongly correlated with quantitative fat infiltration measurements using AI (Fig. 5D; Rs = 0.82, *p* < 0.001).

### Evaluation and example application of fat distribution measurements

Fat fractions calculated from cross-sectional slices displayed good reliability and repeatability (Figs. 4A, 5A, Supplemental Tables 1–3); though, as expected, intra-observer, inter-observer, and AI output-to-vetted, and scan-rescan differences in cross-sectional area and fat fraction were slightly higher than volume differences over the whole muscle. Cross-sectional area differences ranged from 0.94 ± 1.63% (popliteus, intra-observer) to 29.43 ± 45.89% (pectineus, inter-observer). Cross-sectional area differences between AI labels and vetted labels AI label ranged from 3.58 ± 3.46% (vastus lateralis) to 28.37 ± 22.59% (gracilis). Scan-rescan cross-sectional area differences ranged from 2.23 ± 1.12% (vastus lateralis) to 8.73 ± 4.98% (quadratus femoris). The fat fraction calculated at the cross-section level were also relatively consistent; differences ranged from 0.39 ± 0.26% (semimembranosus, intra-observer) to 6.22 ± 9.79% (quadratus femoris, inter-observer). AI-output-vetted fat fraction differences ranged from 0.63 ± 0.51% (phalangeal extensors) to 14.50 ± 11.06% (gracilis). Scan-rescan fat fraction differences ranged from 2.28 ± 1.11% (vastus lateralis) to 8.62 ± 5.01% (quadratus femoris).

**Figure 6:**
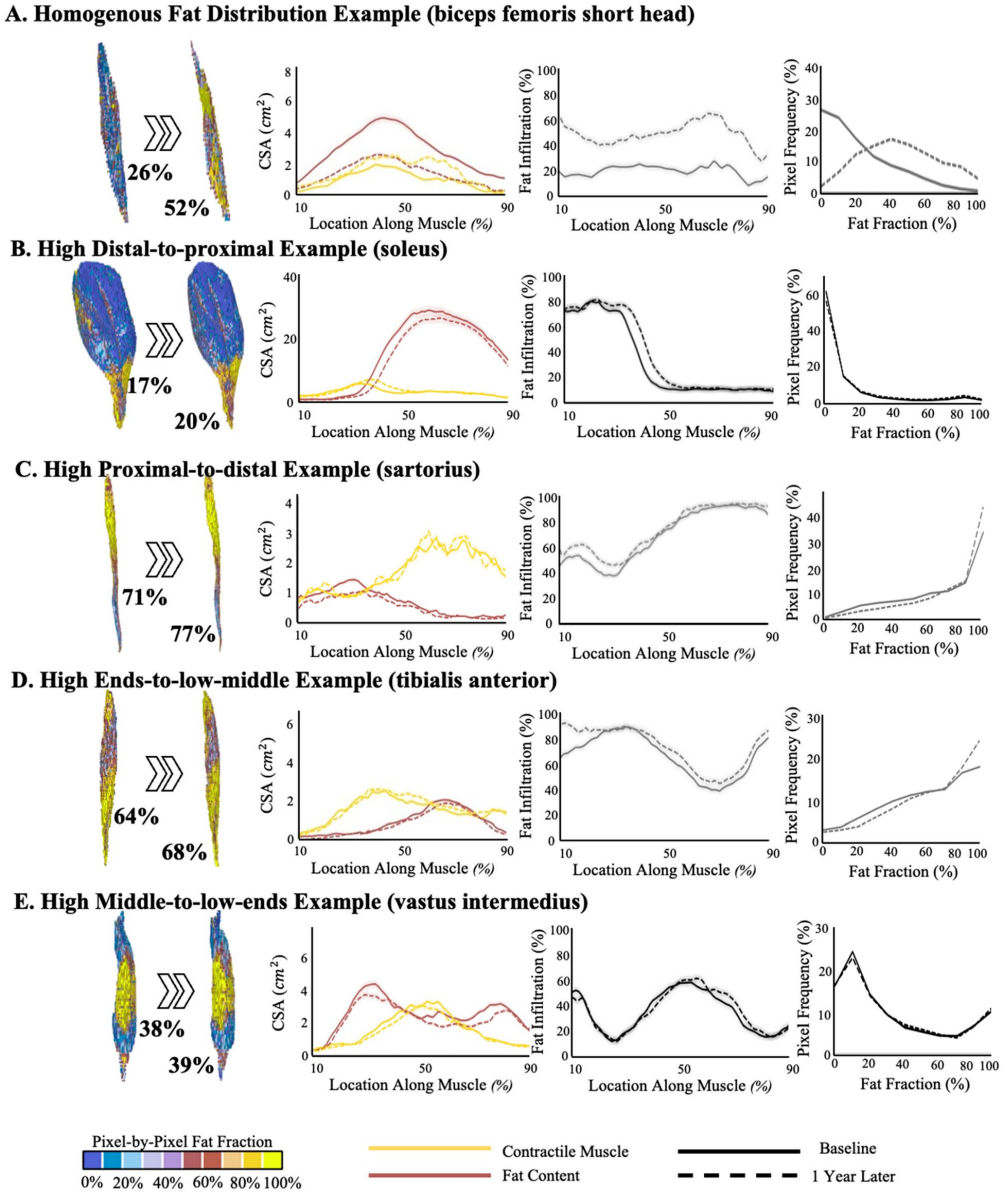
Examples of the five different types of fat distribution and progression for several different muscles. For each type, the right 3D models are the pixel-by-pixel fat fraction, in which each pixel is color coded based on fat fraction (%) from 0% (blue) to 100% (yellow). The left most muscle is at baseline, and the right most is after 1 year. The total fat fraction (%) for the entire muscle volume is given in black. Next, provided is the cross-sectional area (CSA) graphs for contractile muscle (red/pink) and fat (yellow) along the length of the muscle (distal to proximal, %) at baseline (solid line) and 1 year later (dotted line). After, the fat fraction (%) along the CSA is provided as a function of location along the muscle at baseline (solid line) and 1 year later (dotted line). Lastly, the frequency in which each pixel’s fat fraction is found is displayed as a histogram from 0% fat fraction to 100% fat fraction at baseline (solid line) and 1 year later (dotted line). All CSAs are illustrated from 10 to 90% along the muscle to avoid potential over interpretation of change in CSA from small areas at the ends of the muscle. Note—the contractile and fat CSA plots contain a light cloud that indicated ± 5% error; and the fat fraction plot contains a light cloud to indicate ± 2.5% error.

**Figure 7:**
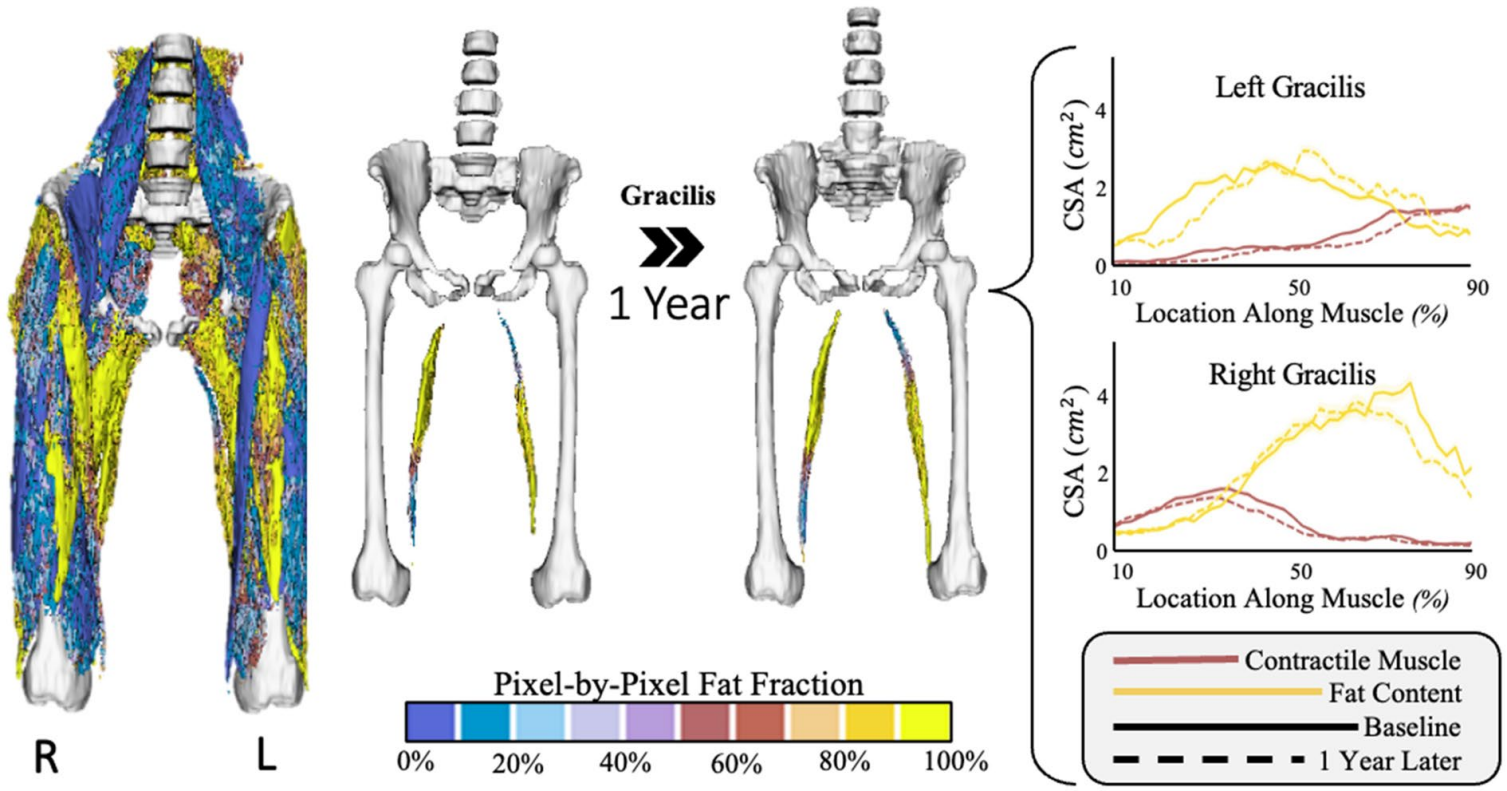
Example patient with opposing fat fraction patterns in the gracilis, specifically, the right gracilis exhibits a “high proximal-to-distal” fat fraction pattern while the left gracilis exhibits a “high distal-to-proximal” pattern. (Left) demonstrated the entire pixel-by-pixel 3D fat fraction model for the entire hip to knee region at baseline, in which each pixel is color coded based on fat fraction (%) from 0% (blue) to 100% (yellow). (Middle) Focused view of the left and right gracilis muscle’s pixel-by-pixel 3D fat fraction model compared from baseline to 1 year later. (Right) The cross-sectional area (CSA) graphs for contractile muscle (red/pink) and fat (yellow) along the length of the left (top) and right (bottom) gracilis (distal to proximal, %) at baseline (solid line) and 1 year later (dotted line).

Representative visualizations and quantifications of muscle and fat distribution demonstrate highly heterogenous patterns of progression across, between, and within muscles (Fig. 6). Some muscles showed relatively homogeneous fat fraction distributions, as evidenced by relatively low variation in fat fraction along their length (Fig. 6A). However, many muscles had greater variation in fat fraction along their length (Fig. 6B–E), with some regions having high fat fraction (> 70%) and other regions exhibiting lower fat fraction (< 20%). Within those muscles, the regions of high and low-fat fraction also varied, including: (1) high distal-to-low proximal (e.g., Fig. 6B), (2) high proximal-to-low distal (e.g., Fig. 6C), (3) high ends-to-low center (e.g., Fig. 6D), and (4) high center-to-low ends (e.g., Fig. 6E). Analysis of the change between baseline and one-year follow up showed that changes in lean muscle volume, fat volume, and fat fraction between time points are most evident in the regions of transition between high and low-fat fraction. Finally, an example analysis of the right and left sides of an individual subject demonstrates that the pattern of fat fraction distribution and associated progression of the same muscle can vary between limbs of the patient (Fig. 7).

### Evaluation and example application of STIR brightness measurements

**Figure 8:**
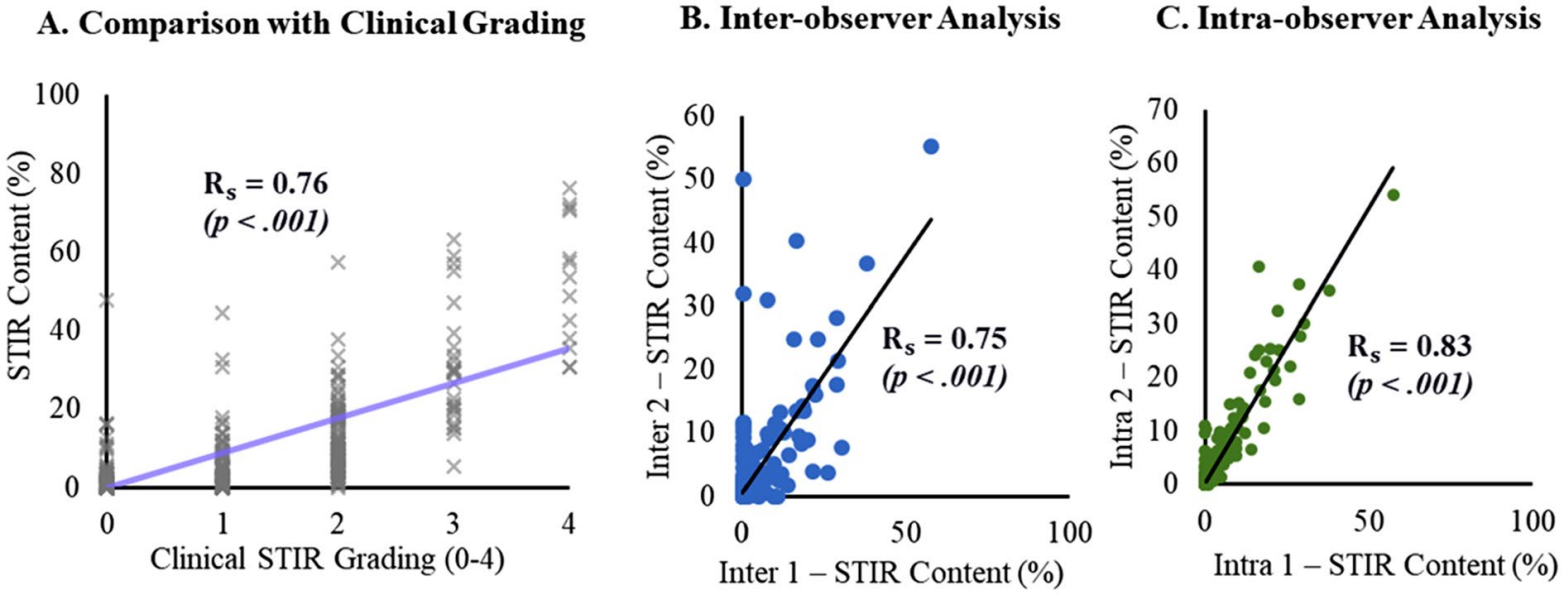
Results for the proposed STIR quantification method. (**A**) Relationship between the clinical STIR grading (0–4) and segmented STIR content (%) across the twenty analyzed muscles in 31 patients. The (**B**) inter-observer and (**C**) intra-observer STIR content (%) results across the twenty-muscles analyzed in 10 patients.

**Figure 9:**
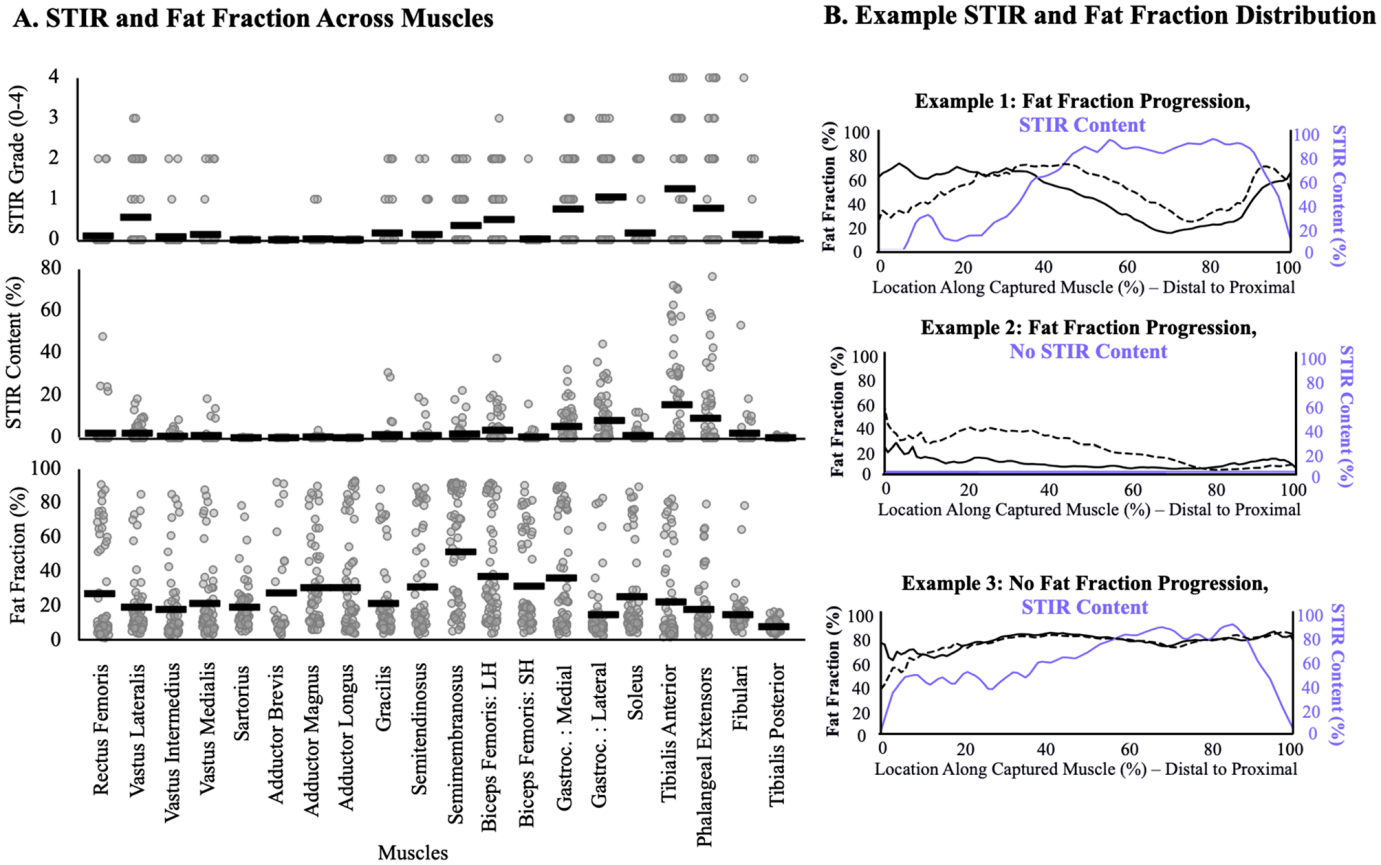
(**A**) Distribution of (top) clinical STIR grading (from 0 to 4), (middle) STIR content (utilizing the proposed method, %), and (bottom) fat fraction (%) for all 31 patients across twenty muscles. Each point is illustrated by a grey dot, and the average is shown as a black horizontal bar. (**B**) Examples of STIR content and fat fraction along the length of the muscle (distal to proximal, %). The examples shown are three different patient’s tibialis anterior, in which the STIR content (purple) found at baseline is displayed as compared to their fat fraction at baseline (solid black line) and fat infiltration 1 year later (dotted black line). Displayed are three different relationships: (top) fat infiltration increases with progression but there is no STIR content at baseline; (middle) fat infiltration increases with progression and there is corresponding STIR content at baseline; (bottom) no fat infiltration increases over time but there is STIR content at baseline.

Quantification of STIR content correlated well with clinical measurements. The Spearman correlation between measured STIR content (%) and clinical ratings (Fig. 8) yielded an Rs = 0.76 (*p* < 0.001). Comparison across and between observers revealed good consistency. The average difference in STIR content (Supplemental Table 4) was 1.39 ± 4.34% between observers and 0.79 ± 2.18% within observers. Example analysis of the distribution of STIR content (Fig. 9) reveal heterogenous patterns of STIR content, across subjects, between muscles, and within muscles. While some muscles demonstrated higher average STIR % across subjects (e.g., tibialis anterior), other muscles demonstrate rare to no STIR % across the study sample (e.g., adductor magnus). By contrast, these same muscles exhibit similar ranges of fat fraction % across the same subjects. Analysis of how STIR content relates to fat fraction progression in three example cases (Fig. 9) reveal that while in some cases, STIR content was elevated in regions that experience fat progression, in other cases fat fraction progression occurs without high STIR content. In other cases, there was minimal fat fraction progression despite the presence of high STIR content.

## Discussion

The pattern and progression of FSHD on muscle involvement is highly variable across patients. The capacity to quantify all individual muscles in their entirety has the potential to: (1) better characterize disease expression and the spatial process of change, (2) identify muscle-specific thresholds of fat replacement that most likely represent an accelerated phase of progression, and (3) estimate what degree of modulation would qualify as a meaningful response to therapy. In the data presented, AI-enabled measures generated from individual muscles in the lower extremities demonstrate high intra- and inter-rater consistency and small amounts of change across a 3-month interval, supporting their functionality as useful biomarkers. As expected, greater analytic variation was observed in small muscles and in muscles having higher levels of fat infiltration where borders are more difficult to identify.

Validation of the AI’s raw label output as compared to its final vetted segmentation was carried out on several scans of varying fat fraction levels (low, moderate, and high). It would be expected that scans with higher fat fractions would be more prone to AI segmentation error due to the difficulty in distinguishing the muscle borders as well as the heterogeneity in fat infiltration presence and its impact on muscle shape. On average across each muscle, the AI performed well, with most Dice similarity coefficients achieving values above 0.90. The muscles that achieved lower dice similarity coefficients (0.60–0.80) were muscles that were smaller and those that typically become more difficult to segment upon fat infiltration (i.e. deep hip muscle, the gracilis, etc.). When compared to current literature, our model performed similarly. For an example, an AI model that segmented individual slices of the muscles of the thigh in patients with FSHD recorded dice coefficient metrics ranging from 0.85 to 0.95 per slice^32^, with similar work reporting a drop in Dice similarity coefficient values with increasing fat presence^33^. AI muscle segmentation in other populations (such as post-menopausal women) demonstrated similar results as well, such as Dice coefficients around 0.82 for muscle segmentation in the hips and thigh^34^. Lastly, muscle segmentation methodology utilizing a non-AI approach (template and registration) achieved comparable results to ours when applied in post-menopausal women (average Dice score of 0.73)^35^. Overall, the most significant differences between the AI model that we utilized as compared to those in current literature are (1) it segments all the muscles of the lower extremity as opposed to only a subset of muscles, and (2) it segments muscles as 3D volumes as opposed to multiple 2D areas, a consideration that ensures disease characterization if fat infiltration or STIR + regions are outside the 2D extent.

Our highest repeatability errors (Fig. 5) occurred in the smallest muscles and in muscles with fat infiltration between 40 and 60%. This indicates that change in fat fraction and or FSHD progression may be harder to accurately capture in those small muscles or muscles in that fat fraction range and pose as a limitation when interpretating fat distribution results as presented here. For studies focused on accurate segmentation of very small muscles that may be fat replaced, increasing pixel-resolution is recommended. Beyond acquisition considerations, we believe more AI training will improve the accuracy of the segmentations in these small muscles and 40 –60% fat fraction regions. The AI model utilized in this work was trained on data collected from 99 FSHD patients, one could imagine that as the AI is trained on more scans of differing FSHD presentation, the AI accuracy will increase and the resultant inter-observer, intra-observer, and 3-month repeat repeatability will significantly improve. The importance of such could indicate that with time even the most complicated of FSHD cases can be segmented with decreasing need to consider potential effects of repeatability when interpreting results.

Some insight can be obtained by comparing these reproducibility values to other studies^11,16,18,24^ and the phenomenon of muscle analytic exclusion. The differences between the 3-month scan repeat measures observed in this study are consistent with general measurement considerations, with smaller muscles showing less repeatability than large muscles. A prior reproducibility study in FSHD over a four month interval has a somewhat similar magnitude of results, though direct comparison is challenging owing to specific regions being composites that include intermuscular tissue (e.g. thighs) and individual muscles having significant numbers of excluded samples that bias estimates (e.g. tibialis anterior only 23/34 included)^24^ All muscles that were imaged generated analyzable data in the current approach. Similar to that study, our use of 3-month interval data leaves open the possibility that some individual subjects/muscles exhibited progression. This is supported by the proportional bias towards lower muscle volume and higher fat fraction in the Bland–Altman scan-rescan comparisons. Two other points warrant mention in terms of expressing fat fraction. First, we investigated the effect erosion of the muscle labels had on fat fraction results, specifically change in fat fraction from 0 to 3 months. We found the change in fat fraction from uneroded and eroded labels were highly correlated (r = 0.89), with no bias in a particular direction. This minimal difference indicates erosion is not required for our data set; however, the chemical shift in the images used in this study was small (roughly ½ pixel). Therefore, we opted to not perform erosion in order to fully capture the detailed, complex patterns of fat infiltration. For other studies in which chemical shifts are higher, it is important to examine the potential impact on fat calculations and consider erosion. Second, as well as overall muscle fat fraction, the literature uses other derived parameters like muscle fat infiltration (MFI) (removing confluent fat regions and reporting the fat within lean muscle tissue)^15^. While it is not surprising that removing confluent fat could reduce measurement variance, in our conceptualization it is not a desirable approach in that: (1) it removes the major component of disease variance (confluent fat) that is increasing over time, and (2) can result in non-overlapping comparisons of anatomical extent (different pixel coverage) across time.

There was strong concordance in the STIR analysis was observed between radiologist ratings and derived STIR content % values for each muscle. On the few occasions where STIR values deviated, we believe the discordance relates to human error. Post hoc examination of these deviations show that most were attributable to brightness at the edges of the slab locations being misclassified, confusion about whether the pixel blush was related to coil sensitivity vs true signal, or simply discordance in rating. With AI, these subjective calls are minimized, and it is also clear that the measure derived pixel-based percentage of STIR + has quantitative advantage over the rating scale that remains categorical (e.g. < 30%, 30–60%, etc.). Other studies have employed histogram^36^ and/or texture analyses^19^—to assess STIR+ regions. While the current application of the AI-based method focused on distinguishing between STIR+ and STIR- pixels and regions, the method could be extended to quantify hyperintense STIR (++) regions. While quantitative methods (such as T2 mapping) also have the potential to assess sub-threshold water features that may have value as biomarkers^7,18,37^, these methods are challenging to implement for large anatomy coverage due to the extensive imaging time and equipment required.

The methods presented here generate significant amounts of data at the muscle-by-muscle level. The next challenge is to determine how to condense these measurements into biomarkers that can be used to test the efficacy of treatments. While fat fraction is the most used MRI-based measure of disease expression, it can be complemented with other MRI-derived metrics. For example, lean muscle volume and fat volume analyzed as individual components can be interrogated for decline (such as in muscle atrophy) or for compensation (lean muscle growth or hypertrophy). Beyond volume, CSA analysis provides both a visually useful index to evaluate where in the muscle changes are occurring, and the ability to readily compare the relationships between tissue components such as fat and STIR intensity. The ability to localize sub-regions of muscles could provide new insight into particularly vulnerable tissues that could predict imminent future disease progression. Extending on this conceptual framework would be leaving the data in 4D pixel-space and using computational models to interrogate change over time^38^.

There are potential limitations of this study that should be addressed. We utilized 3-month data as a reproducibility time-point, but it is possible that some muscles exhibited real change over this interval. While including these values generates conservative estimates for progression, these estimates may reduce sensitivity to identify real changes that occur over this interval. Future work, including more longitudinal samples and evaluating regression curves for each individual muscle, will help determine how to best utilize this baseline and 3-month data to establish thresholds for detectable disease progression.

Data presented here demonstrates tremendous variability in disease involvement across individuals, muscles, within muscles, and across specific muscles that have similar fat fraction/STIR measurements. There are several potential mechanisms that lead to these differences. It remains possible that DUX4 levels could relate in some way to the pattern of fat and STIR+ although this has not been examined to date. Furthermore, differential use and loading of muscles may also influence the pattern of disease expression; a similar concept has been found in lower limb muscles in Duchenne muscular dystrophy^39^. Combining these detailed measurements with other dependent measures, such as genetic biomarkers (allele length, methylation, as examples), detailed measurements of strength that could be mapped onto lean muscle tissue patterns, and complex motion capture to address primary deficit and compensatory features, will help elucidate the features that contribute to heterogeneity in involvement and expression. Further, developing multi-variate models to map individual muscles onto functional tasks (e.g. six-minute-walk test) or patient reported outcomes, will be helpful to better understand disease expression and build personalized models of progression.

Advanced data analytics approaches that incorporate these muscle-level metrics will help determine the most reliable strategy to follow disease progression and the potential for therapeutic modulation over time. At present, three possible strategies exist. One strategy is to create and track a composite score of all measured data. A second strategy, which has been used in clinical trials for FSHD^40^, is to define a specific severity range that encompasses muscles that are thought to most likely progress and follow only those muscles over time. A third strategy, which has been used in natural history studies for Duchenne muscular dystrophy^41^, is to choose a representative muscle and track that muscle over time. In FSHD, it is possible that a fourth approach will be needed, especially the likelihood of any individual muscle across the body progressing substantially over a 1–2-year interval is low due to the slowly progressive nature of the disease. For example, attempts to aggregate all muscles into a single composite may be limited in their ability to reveal a relatively small change in a few muscles over time. Creating an index of muscles at risk may provide greater sensitivity if the categorization is empirical, but additional error variance may result from including muscles that present similarly (e.g. 20% fat at baseline) but have different theoretical change rates (e.g. hypothetically the semimembranosus may progress faster than the vastus lateralis). Including all at-risk muscles for each subject and following this personalized set over time represents the optimal approach in FSHD to define patterns and rates of change.

Despite recognition of the unique patterns of fat and STIR within individual muscles in FSHD, they have remained computationally out of reach in a time efficient, practical utilization. With the developed methods and near-term implementation of the approach to the upper body, a full body mapping of disease biomarkers will be achieved. What is needed next are computational models to address how to reduce these derived data into an integrated whole to better understand the disease, and the development of multivariate models to integrate these MRI measures with strength, functional indexes, and complex motion capture data being collected among ongoing studies and trials in FSHD.

### Supplementary Information

Supplementary Tables.

## Data Availability

Data generated or analyzed during the study are available from the corresponding author by request.

## Abbreviations

FSHD: Facioscapulohumeral muscular dystrophy
MRI: Magnetic resonance imaging
CSA: Cross sectional area
AI: Automatic artificially intelligent algorithm
STIR: Short tau inversion recovery
CSS: Clinical severity score
TE: Echo time
TR: Repetition time
