# Supplementary material for: AI driven analysis of MRI to measure health and disease progression in FSHD
Source: Sci Rep. 2024 Jul 5;14:15462. doi: 10.1038/s41598-024-65802-x (PMC11224366; doi:10.1038/s41598-024-65802-x)
Supplement: Supplementary file 1 — Supplementary Tables. [file 41598_2024_65802_MOESM1_ESM.pdf]

Supplemental Table 1: Average results for the Dice coefficient, absolute volume error (%), absolute fat infiltration difference (%), absolute cross-sectional area (CSA) error (%), and absolute CSA fat fraction difference (%) for interobserver (inter, n = 3), intraobserver (intra, n = 3), and scan-rescan (n=26) comparisons for all muscles.

Muscle Name	Dice Coefficient		Absolute Volume Error (%)			Absolute Fat Fraction Difference (%)			Absolute CSAError (%)			Absolute CSA Fat Fraction Difference (%)		
	N=3	N=3	N=3	N=3	N=26	N=3	N=3	N=26	N=3	N=3	N=26	N=3	N=3	N=26
	Inter	Intra	Inter	Intra	Scan-Rescan	Inter	Intra	Scan-Rescan	Inter	Intra	Scan-Rescan	Inter	Intra	Scan-Rescan
psoas major	0.92 ± 0.11	0.93 ± 0.10	5.08 ± 7.24	1.34 ± 2.01	1.21 ± 0.91	0.89 ± 0.86	0.59 ± 0.95	1.00 ± 1.75	5.13 ± 6.98	4.66 ± 5.51	4.08 ± 3.42	1.66 ± 1.98	1.82 ± 2.68	4.03 ± 3.48
iliacus	0.93 ± 0.09	0.94 ± 0.08	0.98 ± 1.18	1.27 ± 1.8	1.04 ± 0.77	0.82 ± 1.32	0.38 ± 0.52	1.26 ± 3.93	3.01 ± 3.39	4.63 ± 4.83	3.28 ± 2.72	1.55 ± 2.33	1.31 ± 1.63	3.30 ± 2.76
gluteus medius	0.96 ± 0.06	0.96 ± 0.05	0.87 ± 1.07	0.5 ± 0.22	1.35 ± 0.94	0.23 ± 0.19	0.14 ± 0.1	1.15 ± 1.34	3.81 ± 3.47	2.51 ± 2.44	4.04 ± 2.13	0.79 ± 0.74	0.55 ± 0.41	4.03 ± 2.17
gluteus maximus	0.98 ± 0.03	0.99 ± 0.02	0.61 ± 1.00	0.17 ± 0.13	1.03 ± 0.77	0.45 ± 0.74	0.15 ± 0.14	0.71 ± 0.85	2.14 ± 2.07	2.64 ± 4.34	3.29 ± 1.60	0.62 ± 0.77	1.31 ± 2.18	3.16 ± 1.46
gluteus minimus	0.90 ± 0.10	0.92 ± 0.08	4.62 ± 5.57	2.69 ± 3.19	1.69 ± 1.69	0.9 ± 0.71	1.03 ± 1.27	0.97 ± 0.70	5.6 ± 4.12	4.95 ± 5.74	5.40 ± 2.82	2.06 ± 1.96	1.74 ± 2.15	5.33 ± 2.85
piriformis	0.89 ± 0.06	0.91 ± 0.04	4.82 ± 4.52	1.95 ± 1.37	2.38 ± 1.99	1.83 ± 1.2	0.75 ± 0.21	1.52 ± 1.11	10.59 ± 6.28	3.62 ± 2.84	7.97 ± 4.49	3.45 ± 1.98	1.43 ± 1.37	8.08 ± 4.55
gemelli*	0.82 ± 0.06	0.86 ± 0.03	4.37 ± 3.47	3.00 ± 2.6	3.61 ± 3.64	1.6 ± 0.15	1.37 ± 0.92	1.63 ± 1.84	12.61 ± 6.73	6.66 ± 4.2	7.50 ± 4.24	3.8 ± 1.48	2.01 ± 1.71	7.59 ± 4.29
quadratus femoris	0.75 ± 0.27	0.83 ± 0.13	7.62 ± 8.79	3.88 ± 3.12	5.08 ± 5.05	7.19 ± 11.9	2.08 ± 2.97	1.90 ± 2.37	13.57 ± 15.3	4.83 ± 2.98	8.73 ± 4.98	6.22 ± 9.79	2.45 ± 3.2	8.62 ± 5.01
obturator internus	0.81 ± 0.13	0.86 ± 0.06	6.09 ± 8.43	2.9 ± 1.76	2.79 ± 2.52	1.15 ± 1.49	0.3 ± 0.09	1.19 ± 1.89	8.05 ± 3.53	2.86 ± 1.78	5.26 ± 2.00	2.69 ± 2.34	1.33 ± 1.44	5.28 ± 2.04
obturator externus	0.90 ± 0.06	0.90 ± 0.07	2.34 ± 1.88	2.2 ± 2.12	2.54 ± 4.03	0.48 ± 0.52	0.61 ± 0.73	1.72 ± 3.12	5.4 ± 6.1	4.28 ± 2.41	7.66 ± 4.24	2.6 ± 4.05	1.47 ± 2.12	7.64 ± 4.33
pectineus	0.85 ± 0.19	0.90 ± 0.12	1.17 ± 1.2	3.17 ± 4.92	1.77 ± 1.52	0.93 ± 1.52	0.3 ± 0.34	0.78 ± 1.00	29.43 ± 45.89	4.44 ± 5.42	5.73 ± 3.54	4.84 ± 8.04	1.27 ± 1.86	5.73 ± 3.60
tensor fasciae latae	0.91 ± 0.09	0.91 ± 0.10	4.4 ± 6.32	5.67 ± 7.87	2.93 ± 3.27	2.4 ± 2.9	3.09 ± 3.61	1.25 ± 1.34	6.88 ± 7.91	5.46 ± 8.23	5.72 ± 4.62	1.82 ± 1.88	1.56 ± 1.99	5.71 ± 4.72
rectus femoris	0.93 ± 0.07	0.93 ± 0.07	3.7 ± 3.25	3.27 ± 5.4	1.93 ± 1.60	1.53 ± 1.79	1.42 ± 2.12	0.93 ± 1.07	5.69 ± 4.25	3.92 ± 3.79	4.56 ± 2.95	3.87 ± 3.27	3.8 ± 3.34	4.65 ± 2.98
vastus lateralis	0.98 ± 0.03	0.98 ± 0.02	1.39 ± 1.63	0.87 ± 0.71	1.43 ± 0.91	0.89 ± 1.1	0.56 ± 0.58	0.70 ± 0.62	1.66 ± 1.79	1.06 ± 1.04	2.23 ± 1.12	0.95 ± 1.07	0.64 ± 0.7	2.28 ± 1.11
vastus intermedius	0.96 ± 0.04	0.96 ± 0.04	0.83 ± 0.71	1 ± 0.77	1.88 ± 1.32	0.36 ± 0.34	0.26 ± 0.27	1.02 ± 1.87	2.2 ± 1.58	2.45 ± 2.23	5.25 ± 5.04	0.61 ± 0.88	0.74 ± 0.98	5.32 ± 5.13
vastus medialis	0.98 ± 0.02	0.98 ± 0.02	0.78 ± 0.86	0.31 ± 0.27	1.21 ± 0.91	0.33 ± 0.43	0.28 ± 0.25	0.88 ± 0.70	1.31 ± 1.22	1.64 ± 1.56	2.93 ± 1.72	0.51 ± 0.63	0.65 ± 0.79	2.91 ± 1.70
sartorius	0.95 ± 0.05	0.96 ± 0.03	2.93 ± 3.55	1.07 ± 0.36	1.65 ± 1.65	1.56 ± 1.91	0.91 ± 0.6	0.94 ± 0.88	3.63 ± 2.12	2.46 ± 2.29	4.15 ± 2.98	2.56 ± 2.01	1.3 ± 0.77	4.04 ± 2.97
adductor brevis	0.79 ± 0.21	0.86 ± 0.13	3.99 ± 3.22	4.26 ± 3.81	2.39 ± 1.88	2.31 ± 3.86	1.32 ± 1.91	1.28 ± 1.60	6.46 ± 4.47	3.9 ± 3.32	6.80 ± 4.75	3.61 ± 3.19	2.16 ± 2.2	6.84 ± 4.83
adductor magnus	0.93 ± 0.10	0.96 ± 0.05	3.84 ± 6.3	1.3 ± 1.43	1.51 ± 1.06	1.71 ± 2.75	0.84 ± 1.05	0.92 ± 1.09	8.34 ± 7.28	2.18 ± 1.55	5.09 ± 2.26	3.32 ± 3.58	0.59 ± 0.36	5.12 ± 2.28
adductor longus	0.88 ± 0.09	0.91 ± 0.08	3.96 ± 3.94	3.92 ± 3.42	1.83 ± 1.89	0.42 ± 0.35	0.26 ± 0.22	1.16 ± 1.81	6.77 ± 4.88	5.29 ± 5.14	7.07 ± 8.40	1.25 ± 1.14	0.87 ± 0.96	7.16 ± 8.56
gracilis	0.86 ± 0.10	0.88 ± 0.09	5.94 ± 4.96	4.99 ± 5.05	1.69 ± 2.01	4.69 ± 5.37	3.88 ± 4.81	1.46 ± 1.88	9.07 ± 6.31	6.92 ± 5.66	6.71 ± 5.36	6.06 ± 5.05	4.38 ± 4.7	6.61 ± 5.44
semitendinosus	0.93 ± 0.06	0.94 ± 0.05	2.04 ± 2.14	1.36 ± 1.73	1.56 ± 1.31	1.24 ± 1.48	0.77 ± 1.09	1.04 ± 1.20	5.31 ± 1.93	3.61 ± 3.22	6.41 ± 3.60	1.42 ± 1.03	1.27 ± 1.41	6.39 ± 3.65
semimembranosus	0.92 ± 0.06	0.95 ± 0.04	3.24 ± 2.7	0.87 ± 1.09	1.93 ± 1.67	0.22 ± 0.15	0.09 ± 0.06	0.63 ± 0.66	5.88 ± 1.65	2.71 ± 2.2	5.95 ± 2.54	0.96 ± 0.09	0.39 ± 0.26	5.82 ± 2.46
biceps femoris: long head	0.93 ± 0.07	0.94 ± 0.05	2.75 ± 2.94	1.84 ± 1.53	1.87 ± 2.23	0.79 ± 0.74	0.8 ± 1.02	0.96 ± 1.28	5.35 ± 2.48	2.89 ± 1.24	6.79 ± 3.89	1.58 ± 1.77	1.94 ± 2.56	6.54 ± 3.66
biceps femoris: short head	0.90 ± 0.07	0.92 ± 0.06	3.54 ± 3.23	2.14 ± 2.1	2.19 ± 1.63	1.79 ± 2.47	0.66 ± 0.55	1.23 ± 1.47	9.06 ± 2.97	5.42 ± 2.64	6.85 ± 6.55	2.87 ± 1.91	1.88 ± 1.48	6.71 ± 6.62
popliteus	0.94 ± 0.03	0.96 ± 0.01	2.04 ± 1.99	0.16 ± 0.27	N/A	0.35 ± 0.46	0.07 ± 0.12	N/A	4.67 ± 5.13	0.94 ± 1.63	N/A	0.78 ± 0.85	0.32 ± 0.55	N/A
gastrocnemius (medial head)	0.96 ± 0.03	0.98 ± 0.01	2.11 ± 1.93	0.55 ± 0.3	N/A	0.49 ± 0.65	0.12 ± 0.03	N/A	2.84 ± 2.29	2.73 ± 2.71	N/A	0.93 ± 0.72	0.73 ± 0.57	N/A
gastrocnemius (lateral head)	0.97 ± 0.02	0.97 ± 0.03	1.02 ± 0.64	0.85 ± 0.89	N/A	0.54 ± 0.3	0.43 ± 0.49	N/A	2.78 ± 2.65	3.15 ± 3.92	N/A	1.18 ± 1.37	1.44 ± 2.15	N/A
soleus	0.96 ± 0.02	0.97 ± 0.02	1.86 ± 1.08	0.86 ± 0.6	N/A	0.54 ± 0.49	0.25 ± 0.2	N/A	4.98 ± 2.28	3.54 ± 1.13	N/A	1.53 ± 1.02	1.09 ± 0.66	N/A
tibialis anterior	0.96 ± 0.04	0.96 ± 0.05	1.88 ± 1.68	1.82 ± 2.23	N/A	0.55 ± 0.47	0.35 ± 0.43	N/A	6.55 ± 1.53	3.59 ± 3.77	N/A	1.54 ± 0.79	0.96 ± 0.85	N/A
phalangeal extensors	0.96 ± 0.03	0.96 ± 0.05	0.9 ± 0.74	1.23 ± 1.41	N/A	0.14 ± 0.11	0.16 ± 0.17	N/A	1.73 ± 1.84	1.73 ± 1.94	N/A	0.41 ± 0.38	0.44 ± 0.56	N/A
fibulari**	0.95 ± 0.02	0.96 ± 0.03	2.03 ± 1.82	0.9 ± 0.44	N/A	0.45 ± 0.2	0.36 ± 0.21	N/A	3 ± 1.94	1.94 ± 1.31	N/A	0.92 ± 0.15	0.96 ± 0.95	N/A
tibialis posterior	0.97 ± 0.02	0.97 ± 0.01	0.44 ± 0.34	0.57 ± 0.33	N/A	0.06 ± 0.04	0.13 ± 0.11	N/A	2.37 ± 1.07	1.19 ± 0.55	N/A	0.55 ± 0.41	0.26 ± 0.29	N/A
flexor digitorum longus	0.92 ± 0.03	0.94 ± 0.01	3.1 ± 2.60	1.21 ± 0.64	N/A	0.7 ± 0.86	0.13 ± 0.04	N/A	4.85 ± 2.57	3.09 ± 1.12	N/A	1.29 ± 0.53	0.78 ± 0.3	N/A
flexor hallucis longus	0.90 ± 0.06	0.91 ± 0.06	1.59 ± 1.24	1.94 ± 0.44	N/A	1.64 ± 1.84	0.72 ± 0.64	N/A	7.42 ± 2.33	5.13 ± 2.48	N/A	2.37 ± 1.7	1.89 ± 1.69	N/A
quadratus lumborum	0.81 ± 0.20	0.88 ± 0.09	9.03 ± 13.82	4.53 ± 4.78	2.25 ± 2.03	2.16 ± 2.19	0.9 ± 0.38	1.59 ± 1.21	12.36 ± 11.91	7.42 ± 6.73	6.35 ± 2.89	2.52 ± 1.63	1.41 ± 0.77	6.16 ± 2.77

Supplemental Table 2: Individual results for the Dice coefficient, absolute volume error (%), absolute fat infiltration difference (%), absolute cross-sectional area (CSA) error (%), and absolute CSA fat fraction difference (%) for the low, moderate, and high fat fraction interobserver and intraobserver comparisons for all muscles.

Muscle Name	DICE Score						Absolute Volume Error (%)						Absolute Fat Fraction Difference (%)						Absolute Cross Sectional Area Error (%)						Absolute Cross Sectional Area Fat Fraction Difference (%)					
	Low (N=1)		Moderate (N=1)		High (N=1)		Low (N=1)		Moderate (N=1)		High (N=1)		Low (N=1)		Moderate (N=1)		High (N=1)		Low (N=1)		Moderate (N=1)		High (N=1)		Low (N=1)		Moderate (N=1)		High (N=1)	
	Inter	Intra	Inter	Intra	Inter	Intra	Inter	Intra	Inter	Intra	Inter	Intra	Inter	Intra	Inter	Intra	Inter	Intra	Inter	Intra	Inter	Intra	Inter	Intra	Inter	Intra	Inter	Intra	Inter	Intra
psoas major	0.97	0.99	0.99	0.99	0.79	0.82	1.73	0.19	0.12	0.16	13.38	3.66	0.80	0.04	0.08	0.04	1.80	1.68	2.19	0.88	0.10	2.12	13.10	10.99	1.11	0.26	0.01	0.28	3.85	4.92
iliacus	0.99	0.99	0.98	0.98	0.83	0.84	0.35	0.25	0.26	0.20	2.34	3.35	0.02	0.04	0.10	0.12	2.34	0.98	1.29	0.20	0.83	3.92	6.91	9.78	0.18	0.02	0.24	0.77	4.24	3.14
gluteus medius	0.99	0.99	0.99	0.99	0.89	0.90	0.11	0.44	0.41	0.32	2.10	0.74	0.05	0.19	0.21	0.20	0.43	0.03	2.10	0.44	1.52	1.88	7.80	5.20	0.23	0.15	0.52	0.53	1.63	0.96
gluteus maximus	1.00	1.00	1.00	1.00	0.95	0.96	0.04	0.09	0.02	0.10	1.77	0.32	0.04	0.03	0.01	0.11	1.31	0.30	1.15	0.24	0.75	0.04	4.52	7.65	0.17	0.06	0.18	0.04	1.50	3.83
gluteus minimus	0.95	0.98	0.97	0.96	0.78	0.83	2.38	0.45	0.52	1.29	10.97	6.34	0.87	0.12	0.20	0.50	1.62	2.48	2.47	0.43	4.06	3.01	10.27	11.40	1.21	0.13	0.68	0.91	4.30	4.19
piriformis	0.91	0.94	0.93	0.93	0.82	0.86	4.07	1.15	0.73	1.16	9.67	3.53	2.08	0.51	0.52	0.91	2.89	0.84	12.25	3.16	3.64	1.04	15.87	6.67	4.45	1.21	1.17	0.19	4.72	2.90
gemelli*	0.88	0.89	0.83	0.84	0.76	0.84	3.61	1.27	1.34	1.74	8.15	5.99	1.75	0.35	1.46	1.60	1.58	2.15	4.84	5.98	16.64	2.85	16.35	11.16	2.41	0.80	5.35	1.27	3.64	3.97
quadratus femoris	0.92	0.94	0.88	0.87	0.44	0.69	2.40	0.27	2.69	5.72	17.76	5.64	0.27	0.09	0.37	0.66	20.93	5.50	3.25	2.60	6.32	3.67	31.15	8.22	0.44	0.51	0.70	0.70	17.53	6.14
obturator internus	0.86	0.87	0.90	0.91	0.66	0.79	2.40	4.56	0.14	1.06	15.74	3.08	0.41	0.36	0.17	0.34	2.87	0.20	6.04	2.63	5.99	1.21	12.13	4.74	1.33	0.61	1.35	0.39	5.40	2.98
obturator externus	0.93	0.94	0.95	0.95	0.83	0.82	1.72	0.61	0.84	1.39	4.45	4.61	0.27	0.15	0.09	0.23	1.07	1.46	2.54	1.53	1.25	6.04	12.40	5.27	0.41	0.17	0.11	0.33	7.27	3.92
pectineus	0.97	0.97	0.96	0.96	0.63	0.76	0.34	0.23	0.63	0.44	2.54	8.85	0.09	0.09	0.02	0.12	2.69	0.69	3.04	0.16	2.84	2.63	82.42	10.53	0.21	0.11	0.19	0.28	14.13	3.42
tensor fasciae latae	0.97	0.98	0.96	0.95	0.81	0.80	0.88	0.06	0.63	2.28	11.70	14.67	0.77	0.06	0.67	2.13	5.75	7.08	2.71	0.00	1.92	1.45	16.00	14.92	0.77	0.00	0.70	0.89	3.99	3.80
rectus femoris	0.99	0.99	0.93	0.94	0.86	0.85	0.27	0.05	4.09	0.26	6.73	9.50	0.10	0.01	0.95	0.40	3.53	3.86	1.77	0.08	5.10	4.03	10.21	7.65	0.20	0.01	4.95	6.32	6.47	5.06
vastus lateralis	1.00	0.99	0.99	0.99	0.95	0.96	0.17	0.21	0.77	0.78	3.24	1.62	0.11	0.15	0.40	0.30	2.15	1.22	0.39	0.22	0.87	0.74	3.71	2.22	0.16	0.15	0.51	0.32	2.17	1.44
vastus intermedius	0.99	0.99	0.97	0.97	0.92	0.91	0.30	0.12	1.64	1.55	0.55	1.33	0.14	0.07	0.18	0.13	0.75	0.57	1.02	0.00	1.58	2.99	3.99	4.36	0.15	0.00	0.06	0.37	1.62	1.86
vastus medialis	0.99	0.99	0.99	0.99	0.95	0.96	0.14	0.01	0.44	0.37	1.76	0.55	0.06	0.00	0.10	0.33	0.82	0.50	0.56	0.56	0.64	0.93	2.72	3.42	0.10	0.09	0.20	0.30	1.24	1.55
sartorius	0.98	0.98	0.98	0.97	0.89	0.92	0.96	0.68	0.80	1.14	7.03	1.39	0.43	0.25	0.49	1.09	3.77	1.40	1.21	0.82	5.15	1.48	4.52	5.08	0.49	0.42	2.68	1.65	4.50	1.84
adductor brevis	0.98	0.98	0.83	0.88	0.57	0.72	0.27	0.37	5.87	7.99	5.84	4.43	0.01	0.08	6.76	3.52	0.15	0.36	1.83	0.11	6.81	6.30	10.74	5.30	0.19	0.01	6.51	4.41	4.14	2.06
adductor magnus	0.99	0.99	0.98	0.99	0.82	0.90	0.21	0.28	0.19	0.69	11.11	2.93	0.09	0.11	0.16	0.37	4.88	2.05	3.96	0.73	4.32	1.99	16.74	3.82	0.80	0.20	1.74	0.66	7.42	0.92
adductor longus	0.98	0.99	0.84	0.90	0.81	0.84	0.20	0.00	8.06	5.42	3.62	6.33	0.03	0.00	0.69	0.41	0.55	0.36	1.41	0.00	10.94	10.27	7.97	5.60	0.12	0.00	2.40	1.90	1.24	0.72
gracilis	0.97	0.98	0.84	0.85	0.78	0.80	0.91	0.13	10.83	10.21	6.09	4.64	0.38	0.01	10.71	9.27	2.98	2.36	1.83	0.81	13.41	11.99	11.96	7.96	0.33	0.17	9.85	9.45	8.01	3.53
semitendinosus	0.99	0.99	0.93	0.93	0.88	0.90	0.14	0.00	4.36	3.31	1.62	0.76	0.02	0.00	2.89	2.02	0.82	0.30	3.08	0.00	6.42	6.19	6.43	4.65	0.32	0.00	1.58	2.79	2.36	1.03
semimembranosus	0.99	0.99	0.89	0.95	0.88	0.92	0.48	0.14	5.87	0.36	3.38	2.12	0.10	0.06	0.38	0.05	0.17	0.15	4.07	0.27	7.30	4.53	6.26	3.32	1.04	0.09	0.86	0.57	0.99	0.50
biceps femoris: long head	0.99	0.99	0.94	0.95	0.86	0.89	0.10	0.08	2.23	2.67	5.92	2.78	0.04	0.04	1.51	1.96	0.83	0.40	2.93	1.49	7.88	3.33	5.23	3.84	0.28	0.17	3.60	4.88	0.87	0.77
biceps femoris: short head	0.97	0.98	0.89	0.93	0.83	0.86	0.29	0.24	6.75	1.77	3.59	4.40	0.04	0.03	4.61	0.94	0.71	1.01	6.60	2.65	12.36	7.90	8.21	5.72	0.67	0.31	3.83	3.26	4.11	2.07
popliteus	0.96	0.96	0.91	0.95	0.94	0.96	0.00	0.00	3.98	0.00	2.13	0.47	0.00	0.00	0.87	0.00	0.17	0.21	0.00	0.00	10.17	0.00	3.85	2.82	0.00	0.00	1.68	0.00	0.65	0.95
gastrocnemius (medial head)	0.99	0.99	0.95	0.97	0.94	0.97	0.32	0.20	1.86	0.70	4.15	0.74	0.11	0.08	0.13	0.14	1.24	0.14	0.75	0.77	2.47	1.59	5.29	5.82	0.24	0.18	0.89	0.70	1.67	1.31
gastrocnemius (lateral head)	0.99	0.99	0.97	0.99	0.95	0.94	0.52	0.49	1.74	0.20	0.80	1.87	0.21	0.04	0.61	0.28	0.80	0.98	0.65	0.91	1.94	0.86	5.74	7.68	0.25	0.20	0.55	0.20	2.75	3.93
soleus	0.98	0.99	0.94	0.97	0.96	0.95	1.14	0.23	3.11	1.43	1.34	0.92	0.04	0.02	1.01	0.34	0.57	0.40	2.95	2.26	7.44	4.42	4.54	3.94	0.37	0.36	1.91	1.65	2.31	1.25
tibialis anterior	0.99	0.99	0.96	0.98	0.92	0.90	0.34	0.03	1.63	1.12	3.67	4.32	0.05	0.01	0.60	0.20	0.99	0.83	7.89	1.25	6.88	1.59	4.88	7.94	0.76	0.18	2.34	0.85	1.52	1.86
phalangeal extensors	0.99	0.99	0.97	0.98	0.93	0.90	0.21	0.03	0.81	0.89	1.69	2.78	0.02	0.01	0.16	0.13	0.23	0.35	0.08	0.28	1.41	0.98	3.71	3.94	0.00	0.09	0.47	0.15	0.75	1.08
fibulari**	0.98	0.99	0.94	0.97	0.94	0.93	0.58	0.40	4.08	1.17	1.44	1.14	0.65	0.24	0.25	0.24	0.45	0.61	0.94	0.80	4.79	1.65	3.28	3.38	0.81	0.50	0.87	0.32	1.09	2.05
tibialis posterior	0.99	0.98	0.97	0.98	0.96	0.96	0.06	0.81	0.72	0.20	0.53	0.70	0.01	0.06	0.08	0.08	0.08	0.26	1.14	0.74	2.82	1.04	3.14	1.80	0.13	0.08	0.57	0.11	0.95	0.60
flexor digitorum longus	0.95	0.95	0.92	0.94	0.90	0.94	0.90	0.94	2.43	1.94	5.96	0.75	0.15	0.13	0.25	0.17	1.69	0.09	3.42	1.82	3.31	3.93	7.81	3.51	0.86	0.44	1.13	1.02	1.89	0.88
flexor hallucis longus	0.91	0.94	0.95	0.96	0.83	0.84	2.99	2.30	1.17	1.45	0.62	2.07	0.28	0.19	0.90	1.43	3.73	0.55	7.46	4.48	5.07	3.04	9.72	7.87	0.59	0.46	2.55	1.46	3.98	3.75
quadratus lumborum	0.91	0.93	0.93	0.94	0.58	0.78	0.28	1.57	1.85	1.98	24.96	10.05	1.16	0.47	0.65	1.03	4.68	1.20	3.52	3.29	7.66	3.79	25.91	15.19	1.36	0.95	1.83	0.99	4.38	2.30

Supplemental Table 3: Average and individual results for the Dice coefficient, absolute volume error (%), absolute fat infiltration difference (%), absolute cross-sectional area (CSA) error (%), and absolute CSA fat fraction difference (%) for the low, moderate, and high fat fraction raw AI output to vetted label comparison for all muscles.

Muscle Name	DICE Score				Absolute Volume Error (%)				Absolute Fat Fraction Difference (%)				Absolute Cross Sectional Area Error (%)				Absolute Cross Sectional Area Fat Fraction Difference (%)			
	Average (N=3)	Low (N=1)	Moderate (N=1)	High (N=1)	Average (N=3)	Low (N=1)	Moderate (N=1)	High (N=1)	Average (N=3)	Low (N=1)	Moderate (N=1)	High (N=1)	Average (N=3)	Low (N=1)	Moderate (N=1)	High (N=1)	Average (N=3)	Low (N=1)	Moderate (N=1)	High (N=1)
psoas major	0.891 +/- 0.156	0.974	0.99	0.711	6.89 +/- 10.05	1.77	0.43	18.46	1.82 +/- 2.32	0.82	0.17	4.48	8.69 +/- 7.08	5.11	4.12	16.84	3.84 +/- 3.97	1.73	1.38	8.42
iliacus	0.944 +/- 0.073	0.985	0.988	0.859	2.72 +/- 4.01	0.64	0.16	7.34	2.62 +/- 4.47	0.06	0.02	7.78	5.7 +/- 5.63	4.73	0.63	11.75	2.96 +/- 4.48	0.71	0.06	8.12
gluteus medius	0.917 +/- 0.132	0.993	0.993	0.764	3.73 +/- 6.17	0.11	0.22	10.86	1.64 +/- 2.7	0.05	0.12	4.75	8.1 +/- 10.92	3.34	0.36	20.59	2.35 +/- 3.34	0.7	0.16	6.2
gluteus maximus	0.974 +/- 0.042	0.998	0.998	0.925	2.05 +/- 3.51	0.04	0.01	6.11	1.58 +/- 2.69	0.04	0.01	4.69	5.53 +/- 6.46	3.94	0	12.63	2.9 +/- 4.05	1.16	0.01	7.53
gluteus minimus	0.923 +/- 0.076	0.955	0.979	0.836	4.42 +/- 5.24	2.41	0.49	10.37	0.7 +/- 0.6	0.85	0.04	1.2	8.85 +/- 8.77	6.2	1.71	18.63	1.73 +/- 1.41	2.07	0.18	2.94
piriformis	0.893 +/- 0.053	0.913	0.933	0.834	4.32 +/- 3.16	4.14	1.24	7.56	2.56 +/- 2.04	2.13	0.77	4.77	12.58 +/- 4.5	12	8.38	17.34	4.39 +/- 2.04	4.65	2.23	6.28
external rotators	0.83 +/- 0.045	0.866	0.846	0.78	5.96 +/- 4.03	4.93	2.55	10.41	2.15 +/- 0.47	2.16	1.68	2.62	13.33 +/- 7.8	10.26	7.52	22.19	3.68 +/- 0.47	4.19	3.57	3.27
quadratus femoris	0.775 +/- 0.251	0.923	0.916	0.485	5.21 +/- 6.65	2.57	0.28	12.78	1.7 +/- 2.7	0.27	0.01	4.81	7.18 +/- 7.12	5.96	0.75	14.83	4.16 +/- 6.31	1.01	0.04	11.43
obturator internus	0.772 +/- 0.212	0.879	0.91	0.528	8.89 +/- 12.15	2.79	1	22.88	3.76 +/- 6.34	0.14	0.06	11.08	11.42 +/- 11.57	6.54	3.08	24.63	3.86 +/- 5.62	0.83	0.4	10.34
obturator externus	0.757 +/- 0.313	0.921	0.954	0.396	11.68 +/- 17.14	3.34	0.31	31.4	9.12 +/- 15.62	0.19	0.02	27.16	19.04 +/- 20.05	7.88	7.05	42.19	7.64 +/- 12.57	0.46	0.31	22.15
pectineus	0.646 +/- 0.559	0.97	0.967	0.001	16.92 +/- 28.64	0.44	0.33	49.98	11.32 +/- 19.49	0.1	0.04	33.83	17.22 +/- 24.15	5.4	1.25	45	10.3 +/- 17.06	0.72	0.2	30
tensor fasciae latae	0.917 +/- 0.073	0.971	0.947	0.833	2.41 +/- 1.4	0.88	2.74	3.62	1.48 +/- 0.75	0.76	2.25	1.43	7.18 +/- 4.61	5.29	3.81	12.44	3.32 +/- 0.93	2.8	2.77	4.4
rectus femoris	0.906 +/- 0.074	0.989	0.845	0.883	5.13 +/- 5.57	0.27	11.22	3.9	2.58 +/- 3.08	0.1	6.02	1.61	10.24 +/- 5.26	4.2	13.84	12.69	6.49 +/- 4.31	1.54	9.37	8.57
vastus lateralis	0.97 +/- 0.04	0.995	0.991	0.924	2.48 +/- 3.71	0.17	0.51	6.76	1.25 +/- 1.59	0.11	0.55	3.07	3.58 +/- 3.46	1.83	1.34	7.57	1.73 +/- 1.73	0.51	0.98	3.71
vastus intermedius	0.967 +/- 0.036	0.989	0.987	0.926	1.22 +/- 1.7	0.31	0.18	3.19	0.7 +/- 1.06	0.14	0.05	1.92	4.13 +/- 2.14	2.87	2.92	6.6	1.48 +/- 1.31	1.11	0.39	2.94
vastus medialis	0.98 +/- 0.023	0.993	0.993	0.954	1.15 +/- 1.6	0.15	0.29	3	0.22 +/- 0.22	0.06	0.12	0.47	4.26 +/- 0.61	4.09	3.76	4.94	1.04 +/- 0.98	0.42	0.52	2.17
sartorius	0.947 +/- 0.051	0.976	0.975	0.888	3.72 +/- 4.59	0.9	1.24	9.01	3.78 +/- 5.7	0.34	0.63	10.37	8.95 +/- 10.58	3.6	2.11	21.14	8.06 +/- 11.32	1.72	1.33	21.12
adductor brevis	0.808 +/- 0.177	0.977	0.823	0.623	8.42 +/- 10.34	0.27	4.95	20.05	2.7 +/- 3.67	0.01	6.88	1.22	12.13 +/- 9.27	3.78	10.5	22.11	4.36 +/- 3.59	0.22	6.62	6.24
adductor magnus	0.89 +/- 0.088	0.987	0.869	0.815	6.87 +/- 7.81	0.78	15.68	4.14	1.64 +/- 1.26	0.2	2.49	2.24	19.27 +/- 14.76	6.14	35.25	16.42	5.85 +/- 3.62	2.12	6.09	9.34
adductor longus	0.835 +/- 0.16	0.984	0.666	0.855	10.99 +/- 13.23	0.2	25.75	7.02	7.79 +/- 13.05	0.03	22.86	0.49	14.74 +/- 17.37	3.56	34.75	5.9	6.58 +/- 10.03	0.38	18.16	1.21
gracilis	0.704 +/- 0.268	0.97	0.709	0.433	20.69 +/- 25.44	0.86	11.83	49.38	20.62 +/- 20.39	0.37	20.32	41.16	28.37 +/- 22.59	4.37	31.53	49.22	14.5 +/- 11.06	1.99	18.57	22.95
semitendinosus	0.932 +/- 0.052	0.99	0.89	0.916	4.6 +/- 4.1	0.14	8.2	5.46	2.78 +/- 4.1	0.02	7.5	0.84	9.54 +/- 3.08	6.36	12.51	9.74	4.24 +/- 4.23	0.82	8.96	2.94
semimembranosus	0.893 +/- 0.077	0.981	0.863	0.835	4.13 +/- 2.73	1.05	5.13	6.22	0.42 +/- 0.21	0.58	0.18	0.5	13.52 +/- 5.36	8.33	19.03	13.19	2.97 +/- 1.4	2.57	4.53	1.82
biceps femoris: long head	0.924 +/- 0.059	0.99	0.876	0.907	5.22 +/- 5.13	0.1	10.37	5.2	2.08 +/- 3.16	0.04	5.73	0.48	8.75 +/- 4.35	5.77	13.74	6.73	3.64 +/- 4.41	1.09	8.73	1.1
biceps femoris: short head	0.879 +/- 0.084	0.975	0.84	0.822	7.87 +/- 6.45	0.43	11.62	11.58	4.01 +/- 4.37	0.03	8.68	3.3	9.5 +/- 1.8	8.02	8.98	11.5	5.22 +/- 3.79	1.18	8.69	5.8
popliteus	0.938 +/- 0.023	0.961	0.915	0.94	1.96 +/- 1.91	0	3.8	2.08	0.34 +/- 0.46	0	0.87	0.17	6.62 +/- 6.78	0	13.54	6.32	1.13 +/- 1.21	0	2.41	0.99
gastrocnemius (medial head)	0.955 +/- 0.037	0.994	0.92	0.95	2.92 +/- 2.38	0.19	4.53	4.05	0.35 +/- 0.28	0.03	0.47	0.54	6.14 +/- 0.41	5.86	6.61	5.95	2.07 +/- 1.2	0.88	3.28	2.06
gastrocnemius (lateral head)	0.975 +/- 0.015	0.993	0.969	0.96	1.27 +/- 1.13	0.04	1.51	2.27	0.47 +/- 0.49	0.06	0.34	1.02	5.59 +/- 0.86	5.27	4.93	6.56	1.86 +/- 1.36	0.63	1.62	3.33
soleus	0.962 +/- 0.033	0.998	0.934	0.95	2.35 +/- 2.01	0.02	3.42	3.6	0.64 +/- 0.62	0.02	1.27	0.63	6.03 +/- 3.45	2.97	5.36	9.76	1.75 +/- 0.9	0.74	2.46	2.05
tibialis anterior	0.931 +/- 0.079	0.992	0.959	0.84	4.03 +/- 6.39	0.02	0.66	11.4	0.5 +/- 0.41	0.03	0.72	0.77	8.52 +/- 7.65	0.55	9.18	15.81	2.9 +/- 2.61	0.05	3.5	5.16
phalangeal extensors	0.924 +/- 0.095	0.988	0.969	0.81	4.81 +/- 6.97	0.21	1.38	12.83	0.66 +/- 0.92	0.02	0.23	1.72	7.71 +/- 9.36	0.99	3.74	18.41	0.63 +/- 0.51	0.07	0.78	1.05
fibulari**	0.915 +/- 0.103	0.983	0.967	0.8	5.01 +/- 7.35	0.89	0.63	13.5	1.37 +/- 1.58	0.89	0.08	3.13	8.88 +/- 10.43	0.91	5.04	20.69	1.71 +/- 1.14	0.83	1.29	2.99
tibialis posterior	0.915 +/- 0.11	0.99	0.966	0.79	5.08 +/- 7.79	0.01	1.17	14.05	0.23 +/- 0.31	0	0.1	0.59	8.96 +/- 7.81	1.14	8.98	16.76	1.2 +/- 0.91	0.15	1.71	1.74
flexor digitorum longus	0.92 +/- 0.038	0.959	0.917	0.88	4.18 +/- 4.26	0.36	3.41	8.77	0.7 +/- 1.2	0.01	0	2.09	9.81 +/- 3.29	6.4	12.96	10.09	2.13 +/- 1.07	1.02	2.19	3.17
flexor hallucis longus	0.947 +/- 0.042	0.989	0.945	0.91	1.53 +/- 2.07	0.03	0.67	3.89	0.7 +/- 0.83	0.01	0.45	1.63	6.27 +/- 2.55	3.41	8.34	7.05	1.8 +/- 1.07	0.58	2.54	2.3
quadratus lumborum	0.779 +/- 0.254	0.925	0.927	0.487	8.27 +/- 7.34	3.88	4.18	16.74	1.59 +/- 0.27	1.89	1.39	1.49	12.22 +/- 11.44	5.78	5.46	25.42	3.76 +/- 3.65	1.21	2.13	7.94

Supplemental Table 4: Average results for the absolute STIR volume difference (ml) and absolute STIR content difference (%) for interobserver (inter, n = 10) and intraobserver (intra, n = 10) comparisons for all muscles.

Muscle Name	Absolute STIR Volume Difference (ml)		Absolute STIR Content Difference (%)	
	Inter (N=10)	Intra (N=10)	Inter (N=10)	Intra (N=10)
rectus femoris	0.23 ± 0.59	0.33 ± 0.94	0.50 ± 1.40	0.80 ± 2.33
vastus lateralis	3.27 ± 8.03	1.19 ± 2.81	0.75 ± 1.70	0.27 ± 0.59
vastus intermedius	0.05 ± 0.24	0.10 ± 0.35	0.07 ± 0.33	0.09 ± 0.29
vastus medialis	0.52 ± 2.19	0.22 ± 0.93	0.47 ± 2.04	0.20 ± 0.87
sartorius	0.49 ± 1.15	0.49 ± 1.15	1.02 ± 2.68	1.02 ± 2.68
adductor brevis	0.00 ±0.00	0.00 ± 0.00	0.00 ± 0.00	0.00 ± 0.00
adductor magnus	0.07 ± 0.17	0.17 ± 0.44	0.02 ± 0.06	0.08 ± 0.20
adductor longus	0.00 ± 0.00	0.00 ± 0.00	0.00 ± 0.00	0.00 ± 0.00
gracilis	0.04 ± 0.11	0.15 ± 0.46	0.15 ± 0.46	0.57 ± 1.95
semitendinosus	0.32 ± 0.80	0.40 ± 1.22	0.24 ± 0.47	0.31 ± 0.70
semimembranosus	0.67 ± 1.37	0.84 ± 1.53	0.41 ± 0.82	0.58 ± 1.01
biceps femoris: long head	2.08 ± 3.98	2.39 ± 3.72	1.53 ± 2.73	1.91 ± 2.94
biceps femoris: short head	0.32 ± 1.28	0.04 ± 0.08	0.39 ± 1.49	0.05 ± 0.09
gastrocnemius (medial head)	3.07 ± 4.51	1.50 ± 2.17	1.90 ± 2.94	0.96 ± 1.41
gastrocnemius (lateral head)	3.53 ± 4.56	2.43 ± 2.41	3.65 ± 5.66	2.32 ± 3.04
soleus	2.13 ± 6.28	1.80 ± 2.79	0.70 ± 2.13	0.60 ± 0.99
tibialis anterior	9.09 ± 9.95	2.04 ± 2.40	11.41 ± 12.41	2.90 ± 3.35
phalangeal extensors	2.09 ± 3.74	1.39 ± 3.65	3.26 ± 5.56	2.20 ± 5.59
fibulari**	1.07 ± 2.04	0.74 ± 1.24	1.32 ± 2.59	0.90 ± 1.50
tibialis posterior	0.03 ± 0.07	0.07 ± 0.14	0.04 ± 0.10	0.10 ± 0.22
